# Transcriptomic analysis identifies differences in gene expression in actinic keratoses after treatment with imiquimod and between responders and non responders

**DOI:** 10.1038/s41598-021-88424-z

**Published:** 2021-04-22

**Authors:** Megan H. Trager, Emanuelle Rizk, Sharon Rose, Kuixi Zhu, Branden Lau, Benjamin T. Fullerton, Jaya Pradhan, Michael Moore, Ayush C. Srivastava, Giselle Singer, Robyn Gartrell, Rui Chang, Larisa J. Geskin, Yvonne M. Saenger, Gary Goldenberg

**Affiliations:** 1 Department of Dermatology, Columbia University Irving Medical Center, New York, NY USA; 2 Department of Medicine, Division of Hematology/Oncology, Columbia University Irving Medical Center, New York, NY USA; 3 Department of Dermatology, Mount Sinai Hospital, New York, NY USA; 4 Department of Neurology, University of Arizona Health Sciences, Tucson, AZ USA; 5 Department of Pathology, Columbia University Irving Medical Center, New York, NY USA; 6 Department of Surgery, Columbia University Irving Medical Center, New York, NY USA; 7 Department of Pediatrics, Division of Hematology/Oncology, Columbia University Irving Medical Center, New York, NY USA; 8 Herbert Irving Pavilion, 161 Fort Washington Avenue, New York, NY 10032 USA

**Keywords:** Skin cancer, Tumour immunology

## Abstract

The presence of actinic keratoses (AKs) increases a patient’s risk of developing squamous cell carcinoma by greater than six-fold. We evaluated the effect of topical treatment with imiquimod on the tumor microenvironment by measuring transcriptomic differences in AKs before and after treatment with imiquimod 3.75%. Biopsies were collected prospectively from 21 patients and examined histologically. RNA was extracted and transcriptomic analyses of 788 genes were performed using the nanoString assay. Imiquimod decreased number of AKs by study endpoint at week 14 (*p* < 0.0001). Post-imiquimod therapy, levels of CDK1, CXCL13, IL1B, GADPH, TTK, ILF3, EWSR1, BIRC5, PLAUR, ISG20, and C1QBP were significantly lower (adjusted *p* < 0.05). Complete responders (CR) exhibited a distinct pattern of inflammatory gene expression pre-treatment relative to incomplete responders (IR), with alterations in 15 inflammatory pathways (*p* < 0.05) reflecting differential expression of 103 genes (*p* < 0.05). Presence of adverse effects was associated with improved treatment response. Differences in gene expression were found between pre-treatment samples in CR versus IR, suggesting that higher levels of inflammation pre-treament may play a part in regression of AKs. Further characterization of the immune micro-environment in AKs may help develop biomarkers predictive of response to topical immune modulators and may guide therapy.

## Introduction

Actinic Keratoses (AKs) are dysplasias of the epidermis that are associated with an increased risk for squamous cell carcinoma (SCC)^1^. It was estimated that in 2005 over 58 million people in the United States carred a diagnosis of AK^2^. AKs are associated with significant health care expenditure, with some studies estimating costs of over $1 billion per year (92% for physician office visits, 5% for prescription medications)^3,4^.

Untreated, AKs can either remain stable, regress, or progress to become squamous cell carcinoma (SCC). It is difficult to predict whether AK will progress to SCC, but there is a greater than six-fold increase in the risk of developing skin cancers with the presence of AKs^5,6^. It has been reported that SCCs originate in a lesion previously diagnosed as an AK with frequencies ranging between 65 and 97%^4^. In contrast, the estimated rate of progression from AK to SCC is less than 5%^7^. Additionally, AKs have been shown to be markers of field cancerization in excised basal cell carcinoma (BCC), SCC, and malignant melanoma (MM) specimens^8^.

The purpose of treating AKs is two-fold—to decrease risk of progression to SCC and to improve cosmetic appearance. Topical imiquimod is a field treatment for AK shown to lead to initial clearance rates of 85%. Sustained field clearance at 12 months was observed in 73% of patients treated with imiquimod^9^. Imiquimod’s antitumoral activity results from activation of the innate immune system and stimulation of antigen-presenting cells such as dendritic cells and macrophages. Imiquimod is an agonist for toll-like receptor 7, which is commonly involved in pathogen recognition and leads to downstream activation of nuclear factor kappa B and induction of pro-inflammatory Th1 cytokines including interferon alpha, tumor necrosis factor alpha, and various interleukins^10–14^.

Using the nanoString assay, we profiled the gene expression patterns in patients before and after treatment with imiquimod. Consistent with its function, we find that imiquimod diminishes gene expression associated with oncogenesis. We also find that imiquimod decreases expression of genes with immune suppressive function. Further, pre-treatment samples from complete responders have a distinct pattern of gene expression relative to partial and non-responders, suggesting that the pre-existing immune micro-environment in the AK may determine imiquimod response.

## Methods

Tissue collection and initial histological examination were conducted at Icahn School of Medicine at Mount Sinai. The study was approved by the Insitutional Review Board (IRB) at Mount Sinai School of Medicine and was conducted in accordance with relevant guidelines and regulations (https://clinicaltrials.gov/ct2/show/NCT03914417). Informed consent was obtained from all participants and research was performed in accordance with the Declaration of Helsinki. Patients with at least four to eight visible AKs on the face and/or scalp were enrolled after informed consent was obtained. One AK from each patient was biopsied (3 mm punch) at day 0 and sent to pathology for diagnostic confirmation and future transcriptomic analyses. The remaining AKs were photographically documented, and one AK was listed as the target lesion. 21 patients with presumptive AK were enrolled in the study. After histological examination, 2 biopsies were found to contain seborrheic keratosis (SK), and not AK. These patients were therefore excluded from analysis, leaving 19 patients with analyzable pre-treatment biopsies.

Treatment with imiquimod 3.75% was initiated on a short cyclical treatment regimen. Imiquimod was applied daily, 2 weeks on/2 weeks off, for a maximum of 6 weeks (4 weeks of therapy with 2 weeks break) as described in a previous study^15^. The number of AKs was recorded at each follow-up visit. Biopsy of the target lesion was performed at week 14 if it was still present. If the target lesion was no longer visible, a biopsy was done at the site where it was previously located. Our study included 14 patients for whom matched pre and post biopsies were available, and 5 patients with unmatched biopsies (either pre biopsies or post biopsies, but not both).

In accordance with previously established methods, efficacy of treatment was measured by comparing the maximum lesion count during treatment (Lmax) to the number of AKs at week 14, two months after completion of treatment^16^. This allows for measurement of subclinical lesions that are unmasked after treatment with imiquimod. Number of AKs at Lmax and at week 14 was compared using a two-tailed paired t-test. Patients with AKs at the end of the trial were considered to be incomplete responders (IR) and patients without AKs at end of trial were considered to be complete responders (CR).

Pathology review, transcriptomic analyses, and statistical analysis were conducted at Columbia University Irving Medical Center with IRB approval (AAAO2758). All experiments were conducted in accordance with institutional guidelines and regulations. Formalin-fixed paraffin-embedded (FFPE) AK specimen blocks were measured and cut to provide a total of 250 mm^2^ of tissue. RNA was extracted using the miRNeasy FFPE kit (Qiagen) following the manufacturer’s protocol. Extracted RNA was quantitated by Agilent Bioanalyzer with RNA Nano chip assay.

RNA expression levels were profiled using a modified human PanCancer Immune Profiling panel (nanoString), which measures the expression of 770 genes plus 18 additional immune genes that were spiked into the panel, for a total of 788 genes^17^. RNA samples that passed quality and concentration standards were hybridized to target-specific probes and controls in a single tube for 20 h at 65 °C using 100–400 ng of RNA. Target-probe complexes were purified and immobilized on the nCounter prep station. Using the nCounter detection analyzer (nanoString), digital counts for each target RNA were acquired. Finally, nSolver software (nanoString) was used for normalization using housekeeping genes and for paired comparisons of gene expression. The NanoString assay includes RNA spike ins, labeled A-F in decreasing order of concentration, with positive spike in F (POS_F) in the raw data accounting for the lower limit of detection. Thus, transcripts with prohibitively low copy number are excluded (normalized to 0)^18^.

In order to determine whether relevant immune cell populations were in fact present in the AKs, we histologically examined the immune infiltrate present in five untreated AKs. 4 micron slides from pre-treatment biopsies were cut and stained with H&E, CD3 (T lymphocytes; clone LN10; Leica; 1:200 dilution), CD20 (B cells; clone L26; Thermo Fisher; 1:1000 dilution), or CD68 (macrophages; clone KP1; Biogenex; 1:100 dilution).

*p* values comparing the differential gene expression was calculated using the false discovery rate (FDR) correction. Gene expression in pre-treatment AKs was compared between two groups: (1) IR versus CR, (2) all patients with AEs compared to patients without AEs. Pathway analysis was performed as previously published^19^. Hierarchical clustering of genes in heat map was performed using a one minus Pearson correlation.

Given our intial set of genes, we applied pathFinder, a previously developed efficient graphical algorithm^20^, to extract a network from the background ConsensusPathDB (CPDB) signaling pathways. The core of pathFinder is the classical Depth First Search (DFS) algorithm, which expands on the initial input gene set by using genes located in the paths connecting input genes in the background network. Since the background network contains directed and undirected edges, in order to make all the edges in background network directed, each undirected edge was transformed into two directed edges with the same two end nodes but opposite directions. These two edges were not allowed to appear simultaneously in one path.

For every gene in the input list, the DFS explored all paths in the background network that started at that gene. The exploration of a path was stopped if it reached length k (here k = 2 was used) or arrived at a node with no valid child node(s). All nodes along the paths were included in the pathFinder output.

For Key Driver Analysis, we used the R package KDA^21^ (KDA R package version 0.1, available at http://research.mssm.edu/multiscalenetwork/Resources.html). The package first defines a background sub-network by looking for a neighborhood k-step away from each node in the target gene list in the network. Then, stemming from each node in this sub-network, it assesses the enrichment in its k-step (k varies from 1 to k = 3) downstream neighborhood for the target gene list. This algorithm takes the network structure and the location of input genes into consideration and allows us to discover key drivers significantly impacting genes in the input list.

## Results

### Imiquimod significantly decreases number of AKs

**Figure 1:**
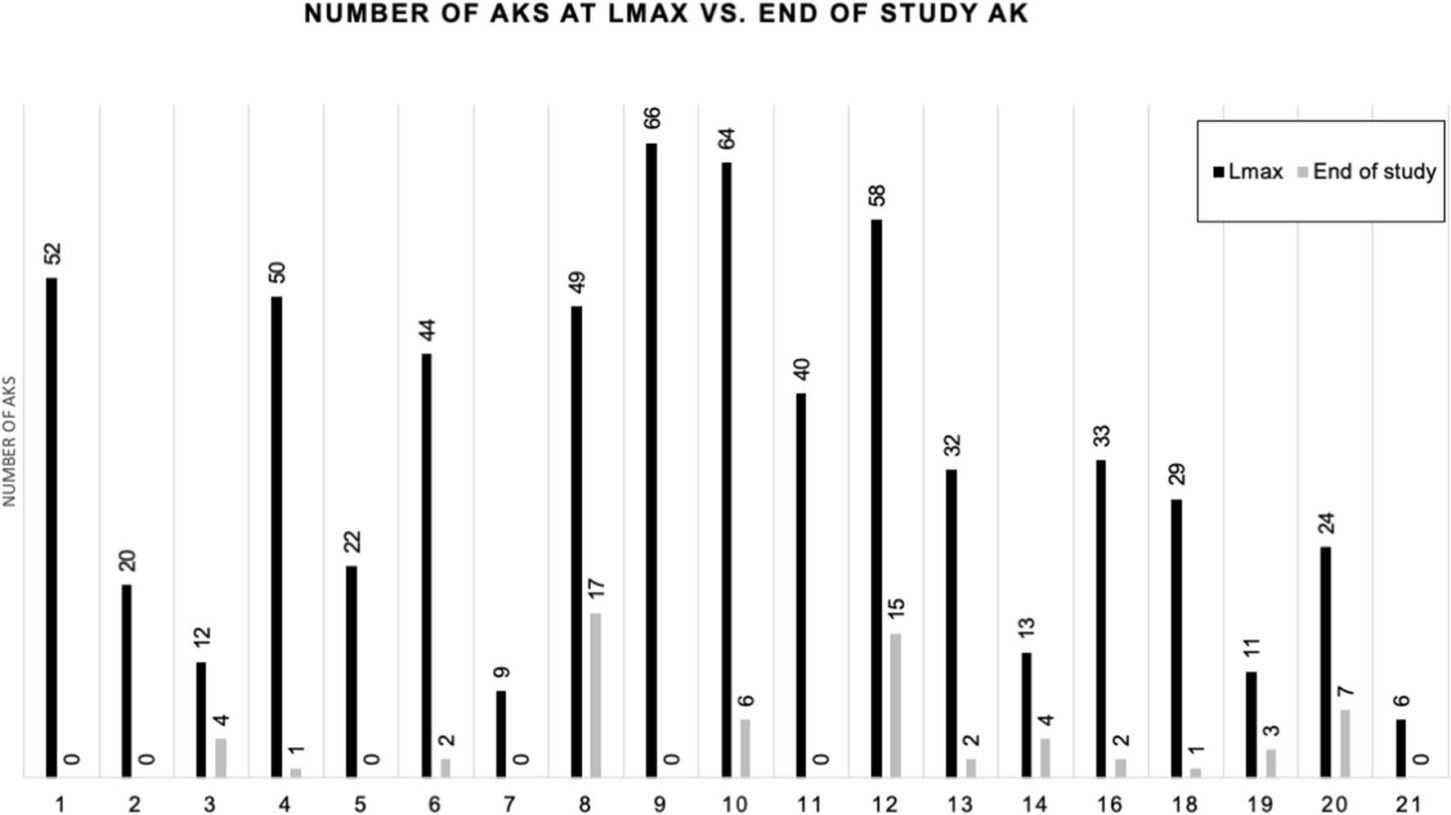
Comparison of maximum number of AKs (Lmax), during treatment with imiquimod (black bars) with number of AKs at week 14, two months post-treatment with imiquimod (grey bars). Numbers above bars indicate number of AKs. Patients 15 and 17 had seborrheic keratoses on initial biopsy and are excluded from this figure. The samples with both pre- and post- treatment biopsies were: 1, 4, 5, 7, 8, 9, 10, 11, 12, 13, 14, 16, 17, 18, 19.

19 patients with confirmed histological diagnosis of AK were included in the final analysis (Table 1). The average age was 73 years and one patient was immunosuppressed, although not a transplant recipient. All patients had a history of prior AK and the treated lesions were located on the scalp (n = 11) and face (n = 8). AEs likely related to use of imiquimod occurred in eight patients, of which six had available pre-treatment biopsies. These AEs included but were not limited to pruritus, severe localized skin reaction, and facial swelling (complete list of AEs included in Table 1). Comparison of Lmax to number of AKs at end of study showed a significant decrease in AKs (t = 7.1, *p* < 0.0001, Fig. 1). Additionally, there was a significant decrease in the total number of AKs by the endpoint of the study at week 14 (t = 6.234, *p* < 0.0001, data not shown).

**Table 1:** Demographic information and treatment response in all patients included in the analysis. *Patients 15 and 17 initial biopsy showed SK and thus are excluded from this table.

Patient	Age	Gender	Immunosuppressed (Yes/No)	History of skin cancer (Yes/No)	History of Smoking (Yes/No)	Treatment area (Scalp/Face)	Complete (C)/Incomplete Responder (I)	Adverse effect
1	68	M	N	N	N	S	C	Left posterior auricular lymphadenopathy
2	85	F	N	N	N	F	C	Pruritus on treatment area
3	68	M	N	N	N	S	I	Pruritus on treatment area, arthralgias
4	78	M	N	N	N	S	I	Flu-like symptoms
5	73	M	N	N	N	S	C	
6	78	M	N	N	N	F	I	
7	77	M	N	N	N	F	C	Viral URI
8	73	M	N	N	N	S	I	
9	77	M	N	N	N	F	C	Flu-like symptoms, facial swelling, gastroenteritis
10	68	M	N	N	N	S	I	
11	67	M	N	N	N	F	C	Viral URI
12	65	M	N	N	N	F	I	Oral apthous ulcer
13	78	M	N	N	N	S	I	
14	73	M	N	N	N	S	I	
16	58	M	N	N	N	F	I	
18	81	M	N	Y (cutaneous SCC)	Y	F	I	
19	83	M	N	N	Y	S	I	
20	75	M	N	N	N	S	I	
21	62	M	Y	N	N	S	C

### Inflammatory genes are downregulated in post-treatment biopsies

**Figure 2:**
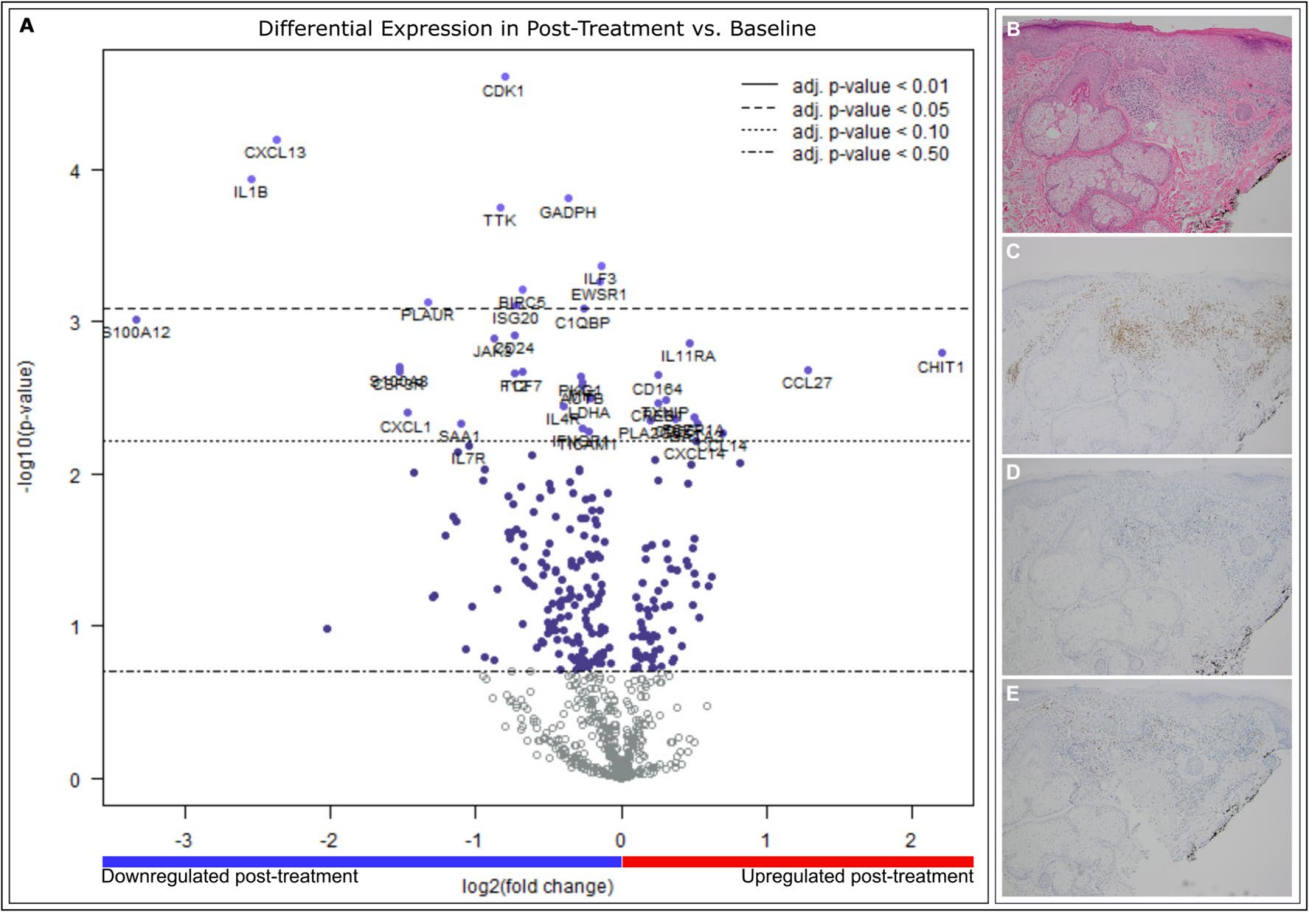
(**A**) Volcano plot of gene expression changes in post versus baseline of pre-treatment samples shows significant downregulation of CXCL13, IL1β, GAPDH, CDK1, TTK, ILF3, EWSR1, PLAUR (*p* < 0.05). (**B**) H&E 10 × magnification of pre-treatment AK shows dense immune infiltration (**C**) IHC staining of CD3 cells at 10 × magnification in pre-treatment AK shows significant staining for CD3 cells (**D**) IHC staining of CD20 cells at 10 × magnification in pre-treatment AK shows few CD20 positive cells (**E**) IHC staining of CD68 cells at 10 × magnification in pre-treatment AK shows moderate staining for CD68 cells.

A total of 32 RNA samples passed quality control checks and were analyzed using nSolver. NanoString results were filtered to search for genes with a change between pre and post-treatment samples (Fig. 2A). Levels of CDK1, CXCL13, IL1B, GADPH, TTK, ILF3, EWSR1, BIRC5, PLAUR, ISG20, and C1QBP were significantly higher in pre-treatment samples than in post-treatment samples (*p* < 0.05; Supplementary Table S1), with CXCL13 and IL1B showing a greater than 2 log2 fold decrease post-treatment. CXCL13 and IL1B are both implicated in both cancer metastasis and recruitment of immune cell populations to tumor cells, particularly B cells and macrophages^22–24^. The general function of these genes, as well as their role in oncogenesis, is described in Supplementary Table S2. Histological examination of five untreated AKs showed significant immune infiltrate of CD3, few CD20 positive cells, and moderate staining for CD68 in untreated AKs (Fig. 2B–E). Of the 16 post-treatment biopsies, 14 were characterized as scar tissue and 2 had residual AK. Thus, post-treatment AKs were not histologically examined. These results suggest that imiquimod diminishes expression of both immune modulatory genes and genes implicated in oncogenic processes.

### Complete responders have a distinct pattern of immune gene expression relative to incomplete responders at baseline

**Figure 3:**
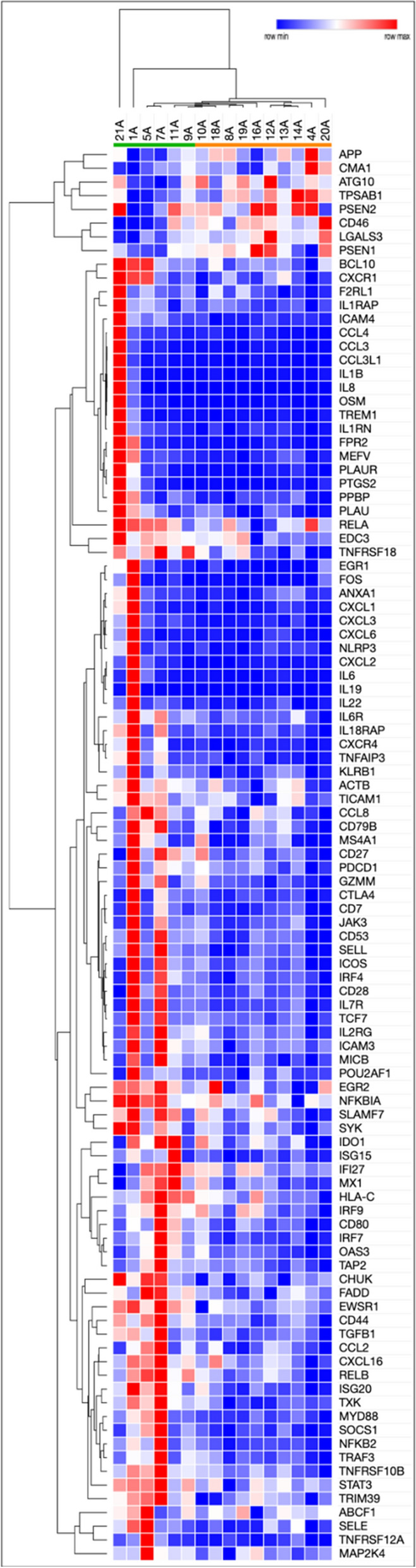
Heatmap of 103 genes differentially expressed between complete responders and incomplete responders. Data shown is pre-treatment gene expression for genes that are differentially expressed between CR and IR. Blue indicates a lower expression, red indicates a higher expression. Heatmap was generated using Morpheus v.1 Software. URL: https://software.broadinstitute.org/morpheus/.

We next sought to identify differences in gene expression between IR and CR using the 788 gene panel. 103 genes were differentially expressed in pre-treatment AKs between IR versus CR, 95 of which were upregulated in CR (*p* values and genes shown in Supplementary Table S3). This was significantly more than was expected out of 788 total genes tested (*p* < 0.001, Fischer’s exact test). Further unsupervised clustering of these 103 genes distinguished CRs (patients 5, 1, 7, 11, and 9) from IRs (patients 19, 10, 14, 13, 12, 8, 4, 18, and 16), as shown using a heatmap in Fig. 3. Pathway analyses found that 15 pathways were significantly differentially expressed between CR and IR (Supplementary Table S4).

### Patients with adverse effects are more likely to respond favorably to treatment and exhibit a distinct pattern of gene expression

**Figure 4:**
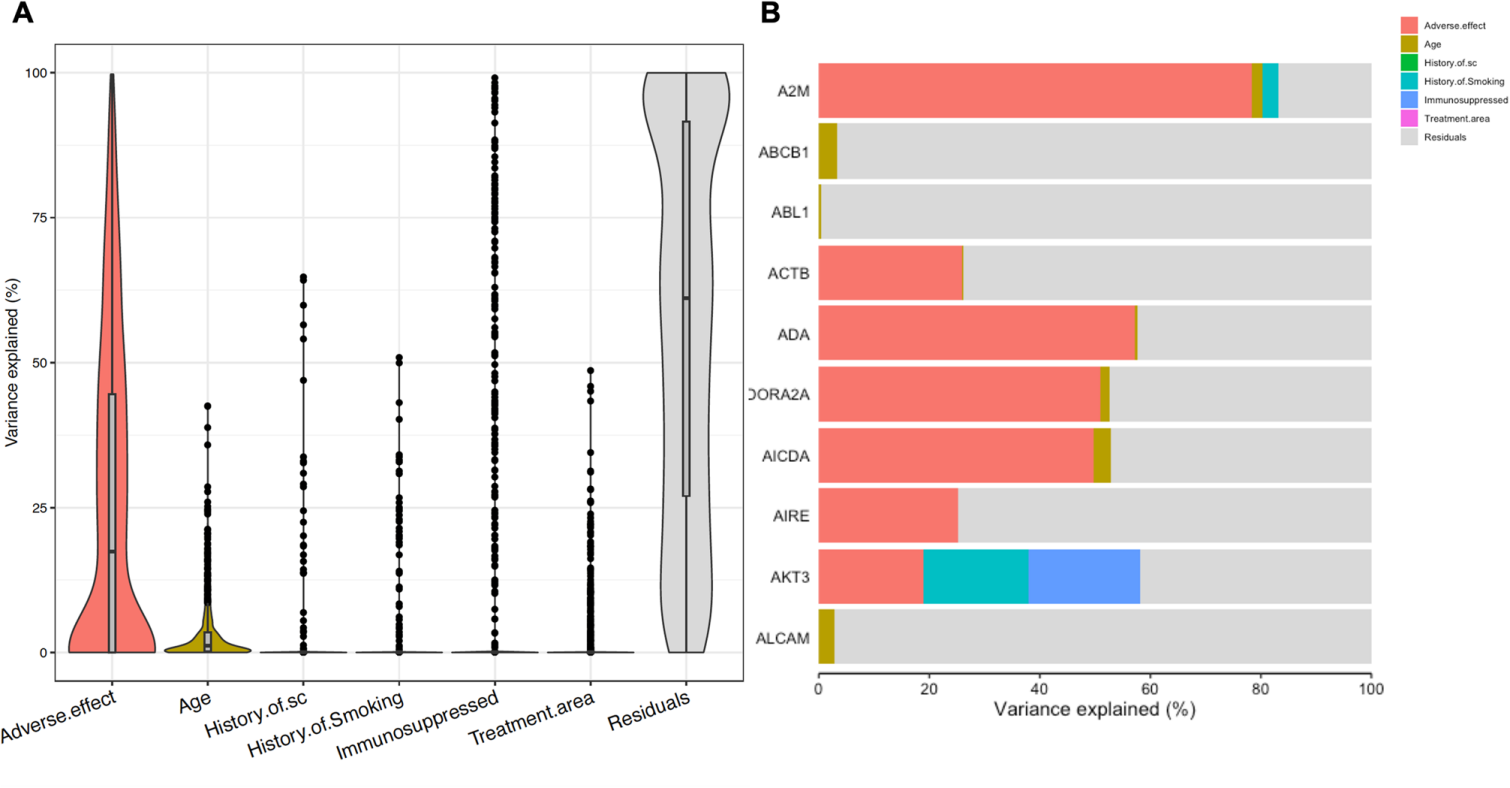
(**A**) Violin plot examining the effects of potential confounders on clinical outcomes. (**B**) Variance explained by gene for the first ten genes in the analysis.

We next examined potential predicters of clinical outcome, including presence of AEs, history of SCC, history of smoking, immunosuppression, treatment area, and age. We found that presence of AEs significantly modulated treatment response, with AEs associated with a more favorable response to treatment (Fig. 4). The top ten most variable genes with respect to AEs are shown in Supplementary Table S5. We then compared patients with available pre-treatment biopsies who had AEs (n = 6) to those who didn’t have AEs (n = 10), finding 104 differentially expressed genes (Supplementary Table S6). 7 pathways were significantly different when comparing patients with AEs to those with no AEs (Supplementary Table S7).

### Network analysis shows densely clustered pathways involved in CR versus IR comparison

Finally, we generated a network from the differentially expressed genes found when comparing CR to IR, patients with AEs to patients without AEs, and CR with AEs to IR without AEs (see methods, Supplementary Figure S1). When comparing these networks, we found that the network utilizing the differentially expressed genes between CR and IR is significantly denser than the other two networks, suggesting that distinct network compartments are enriched by the two sets of differentially expressed genes. To confirm that these differentially expressed genes play a central role in highlighting different biological processes that regulates drug response and adverse effects, we performed pathway analysis. 350 pathways were found to be enriched by the network of CR vs IR, associated with different responses to treatment (Supplementary Table S8) and 4 pathways were found to be associated with the presence of AEs (Supplementary Table S9). 3 pathways overlapped between these two groups (type II interferon signaling, RANKL-RANK, and IL-4).

## Discussion

Approximately 65% of SCCs arise from AKs^7^. Given that imiquimod is a first-line treatment for all AKs, it is important to better understand its effect on gene expression and the immune microenvironment. Further, there has been interest in determining whether gene expression levels can identify high-risk AKs, allowing for treatment of those lesions and not their indolent counterparts. Finally, it has been previously shown that the transcriptomic profile of AKs is more inflammatory and tumorigenic than normal skin^25^, and a better understanding of these differences may help in identifying high-risk AKs.

Our results further support the efficacy of imiquimod for treatment of AKs, given the significant reduction in number of AKs post-treatment relative to Lmax. This is in line with previous studies which have found that imiquimod 3.75% daily application using a short cyclical treatment regimen has similar efficacy as treatment with imiquimod 5% utilizing less frequent application over a longer treatment schedule^15^. We found that presence of AEs including pruritus, facial swelling, apthous ulcer, and flu-like symptoms was associated with improved treatment response.

Our NanoString findings highlight differential regulation of several genes associated with oncogenic pathways after treatment with imiquimod. Housekeeping genes are included in Supplementary Table S10. The genes that were significantly downregulated after treatment with imiquimod (CXCL13, IL1B, GAPDH, TTK, ILF3, EWSR1, BIRC5, PLAUR, ISG20, C1QBP, S100A and CDK1) are all associated with pro-oncogenic and immunoreregulatory pathways^22,24,26–36^. CXCL13, a chemokine ligand for CXCR5 that is associated with B-cell recruitment, may be involved in cancer progression and has been shown to be overexpressed in oral SCC^37,38^. IL1B, GAPDH, and CDK1 in particular have previously been shown to be overexpressed in various SCCs, including lung and head and neck SCC^39,40^. Interestingly, EWSR1 has been shown to inhibit the p53/p21 pathway involved in tumor suppression. Interruption of p53 is also associated with development of cutaneous SCC^41^. As such, the decreased expression of these genes post treatment with imiquimod is likely a reflection of the pre-SCC nature of the AK and of the disappearance of the lesion after treatment with imiquimod. Additionally, imiquimod eliminates the immunosuppressive tumor microenvironment.

Most importantly, we also find differences in pre-treatment gene expression levels between complete and incomplete responders to imiquimod. Of the 103 genes that were differentially expressed between the groups, the majority were upregulated in CR compared to IR (95 upregulated, 8 downregulated).

Further, pathway analysis showed that fifteen pathways were significantly different between CRs and IRs. Several pathways involved in interleukin signaling were significantly different between the CR and IR groups. These include the interleukin 1 receptor pathway, interleukin 6 signaling, interleukin 10 signaling, type 1 interferon and inflammatory cytokines production, and nuclear factor kappa B (NFKB)-related pathways. This suggests that inflammation and cytokine production may predict treatment response to imiquimod. The NFKB pathway has previously been shown to be a therapeutic target for head and neck SCC^42^.

Key driver analyses found three pathways that overlapped as significant key drivers for both response and presence of AEs. These included interleukin 4, receptor of the NFKB ligand (RANKL-RANK) signaling pathway, and type II interferon signaling pathways, all of which are immune-related pathways RANKL-RANK in particular is dysregulated in many types of cancer, including oral SCC, and may be a therapeutic target^43^. The exact role of these pathways on the evolution of AKs in response to imiquimod should be further elucidated using basic science techniques.

Additionally, our results show that presence of AEs may be associated with favorable response to treatment. This is important clinical information, as it highlights the importance of continuing treatment with imiquimod if mild, tolerable AEs occur.

Importantly, the post-treatment biopsies were performed two months after the final treatment with imiquimod. As a result, only 2 of the 16 post-treatment biopsies had residual AK, while the others were characterized as scar tissue. Thus, these gene expression changes are more likely to show the differences between AK and treated skin. Previous studies have examined gene expression changes during treatment with imiquimod and have found upregulation of inflammatory cytokines (IFN-alpha, IL10 receptor 1, TLR7) during treatment^44^. Additionally, overexpression of oncogenic genes and decreased expression of tumor suppressor genes has been observed in both sun-exposed, non-lesional skin and AKs. Treatment with imiquimod reverses this aberrant gene expression^45^. Another study performed biopsies of basal cell carcinomas treated with imiquimod as soon as the tumor showed signs of erosions and similarly found upregulation of genes involved in the inflammatory responses^46^. Our findings differ from these studies due to the later timing of the biopsy. Because the biopsy was performed after treatment and not during treatment, the inflammatory state had likely already resolved, allowing us to examine changes in gene expression between AK and treated skin.

Potential limitations of this research include the small cohort size and the lack of information about progression to SCC. Additionally, the majority of post-treatment biopsies were scar tissue. Analysis of gene expression in treatment-resistant AK may provide more information about resistance to imiquimod. Moreover, as discussed above, a biopsy during treatment with imiquimod may provide additional information regarding mechanisms of clearance.

## Supplementary Information

Supplementary Information 1.

Supplementary Information 2.

## Data Availability

The datasets generated or analyzed during the current study are available from the corresponding author on reasonable request.

## Abbreviations

AKs: Actinic keratoses
SCC: Squamous cell carcinomas
BCC: Basal cell carcinoma
MM: Maligant melanoma
CR: Complete responder
IR: Incomplete responder

## References

[1] Siegel JA, Korgavkar K, Weinstock MA (2017). Current perspective on actinic keratosis: a review. Br. J. Dermatol..

[2] The Lewin Group, I. The Burden of Skin Disease 2005. *Prepared for the Society for investigative Dermatology*.

[3] Bickers DR (2006). The burden of skin diseases: 2004 a joint project of the American Academy of Dermatology Association and the Society for Investigative Dermatology. J. Am. Acad. Dermatol..

[4] Rosen T, Lebwohl MG (2013). Prevalence and awareness of actinic keratosis: barriers and opportunities. J. Am. Acad. Dermatol..

[5] Lee, P. K. in *Clinical Dermatology* (eds Carol Soutor & Maria K. Hordinsky) (McGraw-Hill Education, 2017).

[6] Chen GJ (2005). Clinical diagnosis of actinic keratosis identifies an elderly population at high risk of developing skin cancer. Dermatol. Surg..

[7] Criscione VD (2009). Actinic keratoses: natural history and risk of malignant transformation in the Veterans Affairs Topical Tretinoin Chemoprevention Trial. Cancer.

[8] Lanoue J, Chen C, Goldenberg G (2016). Actinic keratosis as a marker of field cancerization in excision specimens of cutaneous malignancies. Cutis.

[9] Krawtchenko N (2007). A randomised study of topical 5% imiquimod vs. topical 5-fluorouracil vs. cryosurgery in immunocompetent patients with actinic keratoses: a comparison of clinical and histological outcomes including 1-year follow-up. Br. J. Dermatol..

[10] Gorski KS (2006). Distinct indirect pathways govern human NK-cell activation by TLR-7 and TLR-8 agonists. Int. Immunol..

[11] Hemmi H (2002). Small anti-viral compounds activate immune cells via the TLR7 MyD88-dependent signaling pathway. Nat. Immunol..

[12] Reiter MJ, Testerman TL, Miller RL, Weeks CE, Tomai MA (1994). Cytokine induction in mice by the immunomodulator imiquimod. J. Leukoc. Biol..

[13] Del Rosso, J. Q. Topical imiquimod therapy for actinic keratosis: is long-term clearance a realistic benefit? *J. Clin. Aesthet. Dermatol.* **1**(3), 44–47 (2008). PMC3013595 21203362

[14] Gibson SJ (2002). Plasmacytoid dendritic cells produce cytokines and mature in response to the TLR7 agonists, imiquimod and resiquimod. Cell Immunol.

[15] Swanson N (2010). Imiquimod 2.5% and 3.75% for the treatment of actinic keratoses: Results of two placebo-controlled studies of daily application to the face and balding scalp for two 2-week cycles. J. Am. Acad. Dermatol..

[16] Stockfleth E (2014). Reduction in lesions from Lmax: a new concept for assessing efficacy of field-directed therapy for actinic keratosis. Results with imiquimod 3.75%. Eur. J. Dermatol..

[17] Sivendran S (2014). Dissection of immune gene networks in primary melanoma tumors critical for antitumor surveillance of patients with stage II-III resectable disease. J. Invest. Dermatol..

[18] Nanostring User Manual. <https://www.nanostring.com/wp-content/uploads/2020/12/MAN-C0019-08_nSolver_4.0_Analysis_Software_User_Manual.pdf

[19] Hoffman GE, Schadt EE (2016). variancePartition: interpreting drivers of variation in complex gene expression studies. BMC Bioinform..

[20] Samal BB, Eiden LE (2008). pathFinder: a static network analysis tool for pharmacological analysis of signal transduction pathways. Sci. Signal..

[21] Zhang Bin ZJ (2013). Identification of key causal regulators in gene networks. Lect. Notes Eng. Comput. Sci..

[22] Hussain M (2019). CXCL13/CXCR5 signaling axis in cancer. Life Sci..

[23] Biswas S (2014). CXCL13-CXCR5 co-expression regulates epithelial to mesenchymal transition of breast cancer cells during lymph node metastasis. Breast Cancer Res. Treat..

[24] Mantovani A, Barajon I, Garlanda C (2018). IL-1 and IL-1 regulatory pathways in cancer progression and therapy. Immunol. Rev..

[25] Padilla RS, Sebastian S, Jiang Z, Nindl I, Larson R (2010). Gene expression patterns of normal human skin, actinic keratosis, and squamous cell carcinoma: a spectrum of disease progression. Arch. Dermatol..

[26] Hao L (2015). Elevated GAPDH expression is associated with the proliferation and invasion of lung and esophageal squamous cell carcinomas. Proteomics.

[27] Kallakury BV (1997). The prognostic significance of p34cdc2 and cyclin D1 protein expression in prostate adenocarcinoma. Cancer.

[28] Abdullah C, Wang X, Becker D (2011). Expression analysis and molecular targeting of cyclin-dependent kinases in advanced melanoma. Cell Cycle.

[29] Du L (2018). LMO1 functions as an oncogene by regulating TTK expression and correlates with neuroendocrine differentiation of lung cancer. Oncotarget.

[30] Jia R, Ajiro M, Yu L, McCoy P, Zheng ZM (2019). Oncogenic splicing factor SRSF3 regulates ILF3 alternative splicing to promote cancer cell proliferation and transformation. RNA.

[31] Endo A (2016). EWSR1/ELF5 induces acute myeloid leukemia by inhibiting p53/p21 pathway. Cancer Sci..

[32] Cao L (2013). OCT4 increases BIRC5 and CCND1 expression and promotes cancer progression in hepatocellular carcinoma. BMC Cancer.

[33] Narayanaswamy PB (2017). Transcriptomic pathway analysis of urokinase receptor silenced breast cancer cells: a microarray study. Oncotarget.

[34] Gao M (2018). ISG20 promotes local tumor immunity and contributes to poor survival in human glioma. Oncoimmunology.

[35] Kim K (2017). C1QBP is upregulated in colon cancer and binds to apolipoprotein A-I. Exp. Ther. Med..

[36] Bresnick AR, Weber DJ, Zimmer DB (2015). S100 proteins in cancer. Nat. Rev. Cancer.

[37] Kazanietz MG, Durando M, Cooke M (2019). CXCL13 and Its receptor CXCR5 in cancer: inflammation, immune response, and beyond. Front. Endocrinol..

[38] Sambandam Y (2013). CXCL13 activation of c-Myc induces RANK ligand expression in stromal/preosteoblast cells in the oral squamous cell carcinoma tumor-bone microenvironment. Oncogene.

[39] Wu T (2016). Modulation of IL-1β reprogrammes the tumor microenvironment to interrupt oral carcinogenesis. Sci. Rep..

[40] Zhan C (2014). Identification of reference genes for qRT-PCR in human lung squamous-cell carcinoma by RNA-Seq. Acta Biochim. Biophys. Sin. (Shanghai).

[41] Black APB, Ogg GS (2003). The role of p53 in the immunobiology of cutaneous squamous cell carcinoma. Clin. Exp. Immunol..

[42] Lun M (2005). Nuclear factor-kappaB pathway as a therapeutic target in head and neck squamous cell carcinoma: pharmaceutical and molecular validation in human cell lines using Velcade and siRNA/NF-kappaB. Ann. Clin. Lab. Sci..

[43] van Dam PA (2019). RANK/RANKL signaling inhibition may improve the effectiveness of checkpoint blockade in cancer treatment. Crit. Rev. Oncol. Hematol..

[44] Lysa B (2004). Gene expression in actinic keratoses: pharmacological modulation by imiquimod. Br. J. Dermatol..

[45] Torres A (2007). Microarray analysis of aberrant gene expression in actinic keratosis: effect of the Toll-like receptor-7 agonist imiquimod. Br. J. Dermatol..

[46] Urosevic M (2004). Imiquimod treatment induces expression of opioid growth factor receptor: a novel tumor antigen induced by interferon-alpha?. Clin. Cancer Res..

