# Supplementary material for: Transcriptomic analysis identifies differences in gene expression in actinic keratoses after treatment with imiquimod and between responders and non responders
Source: Sci Rep. 2021 Apr 22;11:8775. doi: 10.1038/s41598-021-88424-z (PMC8062619; doi:10.1038/s41598-021-88424-z)
Supplement: Supplementary file 1 — Supplementary Information 1. [file 41598_2021_88424_MOESM1_ESM.docx]

**Supplementary Tables**

Transcriptomic analysis identifies differences in gene expression in actinic keratoses after treatment with imiquimod and between responders and non responders

Short Title: Transcriptomic analysis of actinic keratoses

Megan H. Trager BA*^1^, Emanuelle Rizk BA*^2^, Sharon Rose MD*^3^, Kuixi Zhu MSc*^4^, Branden Lau BS^4^, Ben Fullerton BA^2^, Jaya Pradhan MD^5^, Michael Moore BS^6^, Ayush C. Srivastava MD^2^, Giselle Singer BS^3^, Robyn Gartrell MD^7^, Rui Chang PhD*^4^, Larisa J. Geskin MD*^1^, Yvonne M. Saenger MD*^2^, Gary Goldenberg MD*^3^

**Supplementary Tables: 10**

|  | **Log2Fold Change** | **Standard Error (log2)** | **P-value** |
| --- | --- | --- | --- |
| **CDK1** | -0.798 | 0.16 | 0.0153 |
| **CXCL13** | -2.37 | 0.51 | 0.0197 |
| **IL1B** | -2.54 | 0.573 | 0.0222 |
| **GADPH** | -0.369 | 0.0854 | 0.0222 |
| **TTK** | -0.827 | 0.193 | 0.0222 |
| **ILF3** | -0.139 | 0.0351 | 0.0447 |
| **EWSR1** | -0.153 | 0.0397 | 0.0469 |
| **BIRC5** | -0.677 | 0.177 | 0.0469 |
| **PLAUR** | -1.33 | 0.354 | 0.0469 |
| **ISG20** | -0.722 | 0.193 | 0.0469 |
| **C1QBP** | -0.261 | 0.0703 | 0.0469 |

**Supplementary Table S1.** 11 genes were found to be significantly differentially expressed in post-treatment samples versus a baseline of pre-treatment samples. A negative Log2 Fold change indicates that the gene was more highly expressed in pre-treatment samples. P-values were calculated using the false discover rate (FDR) correction.

| **Gene** | **Function** | **Role in Oncogenesis** |
| --- | --- | --- |
| Cyclin-dependent kinase 1 (CDK1) | Member of the Ser/Thr protein kinase family. Involved in regulation of cell cycle. Phosphorylation of targets by cyclin-bound CDK1 enables cell cycle progression^1^. | Overexpressed in many cancers, including prostate adenocarcinoma and melanoma^2,3^. High activity has been shown to correlate with poor prognosis and increased risk of distant metastasis^4,5^. Overexpressed in laryngeal SCC.^6^ |
| Chemokine ligand 13 (CXCL13) | B lymphocyte chemoattractant that is strongly expressed in the follicles of the spleen, lymph nodes, and Peyer’s patches. Functions in homing of B lymphocytes to follicles. | CXCL13 and its receptor CXCR5 play an important role in many cancers^7,8^. Pathways downstream of the CXCR5 GPCR include the PI3K/Akt, MEK/ERK, and Rac pathways that induce cellular responses in immune cells and cancer cells ^9^. Elevated levels of CXCL13 and/or CXCR5 have been found in the peripheral blood of patients with metastatic cancer ^10^ and are thought to be related to tumorigenesis, tumor cell growth, and metastasis ^7,8^. |
| Interleukin 1β (IL1β) | Member of the interleukin 1 cytokine family. Produced by activated macrophages. Mediator of inflammatory response, involved in cell proliferation, differentiation, and apoptosis. | Found in tumor microenvironment, associated with carcinogenesis ^11^. Implicated in head and neck SCC ^12^. Silencing of the IL-1β inhibited oral SCC growth. |
| Glyceraldehyde-3-phosphate dehydrogenase (GAPDH) | Enzyme that catalyzes the conversion of glyceraldehyde-3-phosphate to 1,3-bisphosphoglycerate in glycolysis | GAPDH is overexpressed in a variety of cancer types, including in cancers of the skin, lung, and breast.^13-16^ In patients with esophageal squamous cell carcinoma, GAPDH expression levels were found to be significantly increased when compared to normal esophageal tissue using qRT-PCR.^17^ These results were found to be applicable to lung squamous cell carcinoma as well.^18^ |
| TTK Protein Kinase (TTK) | Gene encodes protein kinase with ability to phosphorylate tyrosine, serine, and threonine. Essential for chromosome alignment at the centromere during mitosis and is required for centrosome duplication. Critical mitotic checkpoint protein for segregation of chromosomes during mitosis. | Tumorigenesis may occur when the protein fails to degrade and produces excess centrosomes. Associated with development of many types of cancer. Promotes proliferation in bladder cancer.^19^ Targeting of TTK can be used to treat glioblastoma, certain breast cancers, and lung cancers.^20-22^ |
| Interleukin Enhancer Binding Factor 3 (ILF3) | Encodes a double-stranded RNA (dsRNA) binding protein that forms complexes with other proteins, dsRNAs, small noncoding RNAs, and mRNAs to regulate gene expression and stabilize mRNAs. | SRSF3 regulates alternative RNA splicing of ILF3, and increased co-expression of ILF3 isoforms and SRSF3 has been found in several types of cancers.^23^ Associated with progression of renal cell carcinoma.^24^ ILF3 plays oncogenic role in maintaining EGFR-mediated pathway in non-small cell lung cancer.^25^ |
| EWS RNA Binding Protein 1 (EWSR1) | Encodes protein involved in many cellular functions including gene expression, cell signaling, RNA processing and transport. Chromosomal translocations involving EWSR1 gene results in chimeric proteins involved in tumorigenesis. | Associated with pathogenesis of Ewing’s sarcoma.^26^ |
| Baculoviral IAP Repeat Containing 5  (BIRC5) | Member of the inhibitor of apoptosis gene family. Encodes negative regulatory proteins that prevent apoptotic cell death. Gene expression is high in most tumors. | BIRC5 has been found to be mapped to tumorigenic pathways.^27^ Overexpressed in lung cancer tissues.^28^ Associated with worse prognosis in adenocarcinoma^29^. |
| Plasminogen Activator, Urokinase Receptor (PLAUR) | Involved in cell-surface plasminogen activation and localized degradation of the extracellular matrix. | Shown to have a role in metastasis and promoting tumor progression. HMGA1 influences components of the plasminogen activating system including PLAUR and promotes cancer progression.^30^ |
| Interferon Stimulated Exonuclease Gene 20 (ISG20) | Diseases associated include hepatitis C and yellow fever. Related pathways involved the innate immune system and interferon gamma signaling. | IFN gamma found to be involved in promoting cancer immunoevasion.^31^ IFN gamma leads to activation of the JAK/STAT pathway. IFN gamma may have an antiproliferative effect in melanoma and may be associated with decreased invasiveness.^32^ |
| Complement C1q Binding Protein (C1QBP) | Associates with C1r and C1s to form the first component of the serum complement system. Involved in inflammation and infection, ribosome biogenesis, protein synthesis in the mitochondria, regulation of apoptosis, transcriptional regulation, and pre-mRNA splicing. | C1q receptors (cC1aR/CR and gC1qR) play a differential role in carcinogenesis. gC1qR promotes tumor survival and cC1qR expression tags cells for destruction by macrophages.^33^ |

**Supplementary Table S2.** General and cancer-related functions of the genes found to be differentially expressed in post-treatment samples relative to a baseline of pre-treatment.

|  | **logFC** | **AveExpr** | **t** | **P.Value** | **adj.P.Val** |
| --- | --- | --- | --- | --- | --- |
| **CCL4** | -93.72723 | 149.3838 | -4.783812 | 0.000821 | 0.322435 |
| **EWSR1** | -338.5619 | 2717.078 | -4.282722 | 0.001743 | 0.322435 |
| **TPSAB1** | 4186.423 | 6780.273 | 4.188261 | 0.002017 | 0.322435 |
| **BCL10** | -367.2134 | 1215.774 | -4.179648 | 0.002044 | 0.322435 |
| **ISG20** | -222.8004 | 240.3981 | -4.088798 | 0.002355 | 0.322435 |
| **RELB** | -96.65166 | 215.3206 | -3.868732 | 0.003334 | 0.322435 |
| **PTGS2** | -197.8874 | 146.015 | -3.810767 | 0.003657 | 0.322435 |
| **TICAM1** | -115.479 | 384.6744 | -3.647716 | 0.004756 | 0.322435 |
| **F2RL1** | -116.0921 | 515.25 | -3.535371 | 0.005709 | 0.322435 |
| **CD44** | -4366.075 | 13602.83 | -3.481185 | 0.006239 | 0.322435 |
| **TNFAIP3** | -825.0695 | 814.3038 | -3.472931 | 0.006324 | 0.322435 |
| **LGALS3** | 2930.597 | 6054.646 | 3.465214 | 0.006404 | 0.322435 |
| **CXCL16** | -198.6818 | 777.145 | -3.440757 | 0.006666 | 0.322435 |
| **CXCL3** | -105.3029 | 54.40813 | -3.437365 | 0.006704 | 0.322435 |
| **SELE** | -169.955 | 117.7094 | -3.403934 | 0.007083 | 0.322435 |
| **IL6** | -116.4151 | 36.56438 | -3.378593 | 0.007385 | 0.322435 |
| **CXCL2** | -588.9207 | 208.8425 | -3.32499 | 0.008068 | 0.322435 |
| **TXK** | -27.94555 | 48.95 | -3.299776 | 0.008412 | 0.322435 |
| **PLAU** | -289.9132 | 555.0388 | -3.297166 | 0.008449 | 0.322435 |
| **IL1RN** | -258.1977 | 663.7581 | -3.29496 | 0.00848 | 0.322435 |
| **TNFRSF10B** | -83.60049 | 267.7731 | -3.286967 | 0.008593 | 0.322435 |
| **IL6R** | -202.1113 | 385.5088 | -3.253215 | 0.009088 | 0.325506 |
| **HLA-C** | -2713.851 | 5263.796 | -3.191988 | 0.010062 | 0.344729 |
| **EGR1** | -1149.615 | 685.7063 | -3.107017 | 0.011595 | 0.356868 |
| **EDC3** | -98.6437 | 886.4306 | -3.055836 | 0.012632 | 0.356868 |
| **PDCD1** | -33.18325 | 37.1425 | -3.040787 | 0.012954 | 0.356868 |
| **ATG10** | 83.57606 | 230.8313 | 3.022537 | 0.013357 | 0.356868 |
| **CD27** | -218.4002 | 199.1281 | -3.012275 | 0.013589 | 0.356868 |
| **CCL8** | -64.07859 | 77.77375 | -2.960647 | 0.01482 | 0.356868 |
| **IRF4** | -378.1897 | 342.4694 | -2.941582 | 0.015303 | 0.356868 |
| **TNFRSF18** | -131.4898 | 269.6325 | -2.932492 | 0.015538 | 0.356868 |
| **CXCR4** | -1954.994 | 1279.574 | -2.919205 | 0.01589 | 0.356868 |
| **ISG15** | -196.7768 | 228.175 | -2.859124 | 0.017582 | 0.356868 |
| **PLAUR** | -430.3295 | 423.2206 | -2.848117 | 0.017911 | 0.356868 |
| **ACTB** | -15156.3 | 56357.08 | -2.836226 | 0.018274 | 0.356868 |
| **TNFRSF12A** | -226.2175 | 181.7638 | -2.804688 | 0.019272 | 0.356868 |
| **CCL3L1** | -109.4567 | 200.2888 | -2.796962 | 0.019525 | 0.356868 |
| **CCL3** | -104.5354 | 160.1869 | -2.792712 | 0.019666 | 0.356868 |
| **CXCL1** | -1115.391 | 496.5981 | -2.789957 | 0.019757 | 0.356868 |
| **OSM** | -85.68613 | 102.2581 | -2.787544 | 0.019838 | 0.356868 |
| **MYD88** | -531.916 | 1194.759 | -2.785275 | 0.019914 | 0.356868 |
| **ICAM4** | -25.79893 | 43.91438 | -2.779902 | 0.020095 | 0.356868 |
| **IRF7** | -302.5292 | 488.4219 | -2.775165 | 0.020257 | 0.356868 |
| **PSEN2** | 60.98639 | 283.1044 | 2.772982 | 0.020331 | 0.356868 |
| **EGR2** | -428.8516 | 875.4563 | -2.742058 | 0.021421 | 0.356868 |
| **ABCF1** | -147.785 | 1154.424 | -2.740978 | 0.02146 | 0.356868 |
| **SOCS1** | -200.9604 | 264.6338 | -2.732688 | 0.021763 | 0.356868 |
| **CXCR1** | -56.78373 | 70.295 | -2.702993 | 0.022883 | 0.356868 |
| **CHUK** | -100.511 | 671.7175 | -2.692286 | 0.0233 | 0.356868 |
| **GZMM** | -56.70198 | 57.47625 | -2.677375 | 0.023895 | 0.356868 |
| **FPR2** | -113.1852 | 60.8125 | -2.670461 | 0.024176 | 0.356868 |
| **SYK** | -190.4126 | 588.6544 | -2.664708 | 0.024412 | 0.356868 |
| **CTLA4** | -396.4917 | 264.0038 | -2.662215 | 0.024515 | 0.356868 |
| **CD28** | -96.74225 | 73.94813 | -2.623228 | 0.026184 | 0.356868 |
| **CD7** | -130.4743 | 87.55 | -2.612268 | 0.026674 | 0.356868 |
| **IL1RAP** | -124.2772 | 571.2188 | -2.609584 | 0.026795 | 0.356868 |
| **MICB** | -123.7641 | 155.9956 | -2.594049 | 0.027508 | 0.356868 |
| **IDO1** | -135.0206 | 179.685 | -2.568077 | 0.028742 | 0.356868 |
| **TREM1** | -188.894 | 194.3531 | -2.566577 | 0.028815 | 0.356868 |
| **PPBP** | -30.92984 | 37.35 | -2.561194 | 0.029078 | 0.356868 |
| **SELL** | -297.3527 | 210.1756 | -2.556965 | 0.029287 | 0.356868 |
| **TRIM39** | -28.71059 | 150.4075 | -2.54849 | 0.029709 | 0.356868 |
| **RELA** | -52.10477 | 321.895 | -2.547995 | 0.029734 | 0.356868 |
| **FOS** | -9354.32 | 3183.776 | -2.524639 | 0.030931 | 0.356868 |
| **ICAM3** | -251.5484 | 488.4069 | -2.509061 | 0.031755 | 0.356868 |
| **STAT3** | -1802.405 | 8387.961 | -2.507343 | 0.031847 | 0.356868 |
| **MAP2K4** | -125.5951 | 624.1438 | -2.495856 | 0.032471 | 0.356868 |
| **IRF9** | -1062.09 | 3196.687 | -2.489821 | 0.032804 | 0.356868 |
| **NFKBIA** | -1087.733 | 4749.191 | -2.488511 | 0.032876 | 0.356868 |
| **CXCL6** | -85.04975 | 34.4775 | -2.488123 | 0.032898 | 0.356868 |
| **IL1B** | -367.0301 | 498.0056 | -2.480396 | 0.03333 | 0.356868 |
| **NFKB2** | -309.1534 | 647.9844 | -2.47865 | 0.033428 | 0.356868 |
| **ICOS** | -114.836 | 91.04 | -2.475188 | 0.033624 | 0.356868 |
| **PSEN1** | 161.2644 | 1002.704 | 2.454308 | 0.03483 | 0.356868 |
| **IL8** | -4419.473 | 4299.304 | -2.45324 | 0.034893 | 0.356868 |
| **MS4A1** | -87.4864 | 74.16813 | -2.449755 | 0.035099 | 0.356868 |
| **KLRB1** | -124.6993 | 125.4825 | -2.449501 | 0.035114 | 0.356868 |
| **TCF7** | -359.5764 | 401.0344 | -2.445951 | 0.035325 | 0.356868 |
| **IL19** | -471.3497 | 96.18188 | -2.437311 | 0.035843 | 0.35727 |
| **APP** | 1457.008 | 6710.59 | 2.430273 | 0.036271 | 0.35727 |
| **MX1** | -1217.086 | 1583.464 | -2.409694 | 0.037552 | 0.36515 |
| **SLAMF7** | -84.32454 | 151.9275 | -2.399755 | 0.038186 | 0.36515 |
| **CD53** | -655.527 | 744.1756 | -2.390322 | 0.038798 | 0.36515 |
| **CD80** | -45.52112 | 64.9975 | -2.388173 | 0.038938 | 0.36515 |
| **FADD** | -40.35744 | 132.6925 | -2.381355 | 0.039388 | 0.36515 |
| **MEFV** | -31.33797 | 30.26438 | -2.374376 | 0.039854 | 0.365171 |
| **IL7R** | -1041.509 | 697.2619 | -2.357608 | 0.040995 | 0.371308 |
| **CD46** | 768.6986 | 3800.979 | 2.349988 | 0.041524 | 0.371826 |
| **POU2AF1** | -24.79052 | 22.595 | -2.335712 | 0.042533 | 0.372029 |
| **TRAF3** | -147.0682 | 590.1531 | -2.334741 | 0.042602 | 0.372029 |
| **ANXA1** | -8030.69 | 9529.087 | -2.300096 | 0.045157 | 0.372029 |
| **NLRP3** | -102.5554 | 83.99313 | -2.29705 | 0.045388 | 0.372029 |
| **CMA1** | 173.2533 | 311.8956 | 2.294874 | 0.045554 | 0.372029 |
| **TGFB1** | -463.8901 | 986.0625 | -2.294599 | 0.045575 | 0.372029 |
| **IFI27** | -2995.466 | 6202.103 | -2.289363 | 0.045978 | 0.372029 |
| **IL22** | -25.31971 | 13.79188 | -2.278558 | 0.046819 | 0.372029 |
| **TAP2** | -412.1224 | 878.285 | -2.274839 | 0.047112 | 0.372029 |
| **OAS3** | -347.0809 | 620.8638 | -2.265641 | 0.047845 | 0.372029 |
| **IL2RG** | -430.8974 | 530.2794 | -2.261377 | 0.048188 | 0.372029 |
| **CD79B** | -97.05678 | 111.32 | -2.251909 | 0.048959 | 0.372029 |
| **IL18RAP** | -69.97806 | 62.26 | -2.251439 | 0.048997 | 0.372029 |
| **CCL2** | -408.0676 | 648.86 | -2.244614 | 0.049561 | 0.372029 |
| **JAK3** | -313.3058 | 319.6506 | -2.243806 | 0.049628 | 0.372029 |

**Supplementary Table S3.** 103 genes were found to be significantly differentially expressed in incomplete responders (IR) versus a baseline of complete responders (CR). A negative log fold change indicates that the gene is more highly expressed in CR relative to IR.

|  | **Pathway** | **External ID** | **Source** | **P value** | **FDR** |
| --- | --- | --- | --- | --- | --- |
| 1 | signal transduction through il1r | il1rpathway | BioCarta | 0.01422127 | 1 |
| 2 | Interleukin-10 signaling | WP4063 | Wikipathways | 0.01797542 | 1 |
| 3 | DEx/H-box helicases activate type I IFN and inflammatory cytokines production | R-HSA-3134963 | Reactome | 0.01852971 | 1 |
| 4 | cd40l signaling pathway | cd40pathway | BioCarta | 0.02051293 | 1 |
| 5 | Role Altered Glycolysation of MUC1 in Tumour Microenvironment | WP4480 | Wikipathways | 0.02060296 | 1 |
| 6 | RIP-mediated NFkB activation via ZBP1 | R-HSA-1810476 | Reactome | 0.02080113 | 1 |
| 7 | Photodynamic therapy-induced NF-kB survival signaling | WP3617 | Wikipathways | 0.02680811 | 1 |
| 8 | TNF related weak inducer of apoptosis (TWEAK) Signaling Pathway | WP2036 | Wikipathways | 0.02692241 | 1 |
| 9 | Alternative NF-kappaB pathway | nfkappabalternativepathway | PID | 0.03205616 | 1 |
| 10 | IL-6 signaling | None | INOH | 0.03227553 | 1 |
| 11 | Toll-like Receptor Signaling | WP3858 | Wikipathways | 0.03858009 | 1 |
| 12 | IL23-mediated signaling events | il23pathway | PID | 0.03888631 | 1 |
| 13 | pertussis toxin-insensitive ccr5 signaling in macrophage | ccr5pathway | BioCarta | 0.04293296 | 1 |
| 14 | bone remodeling | ranklpathway | BioCarta | 0.04584503 | 1 |
| 15 | ZBP1(DAI) mediated induction of type I IFNs | R-HSA-1606322 | Reactome | 0.0479592 | 1 |

**Supplementary Table S4.** 15 pathways were found to be significantly differentially expressed in CR relative to IR.

|  | **Adverse effect** | **History of SCC** | **History of Smoking** | **Immunosuppressed** | **Treatment Area** | **Age** | **Residuals** | |
| --- | --- | --- | --- | --- | --- | --- | --- | --- |
| IL19 | 0.9976962 | 0.00E+00 | 2.72E-04 | 1.85E-03 | 0.00E+00 | 8.48E-05 | | 0.00010091 |
| NCAM1 | 0.9772503 | 0.00E+00 | 3.53E-12 | 8.46E-03 | 0.00E+00 | 3.62E-05 | | 0.01424909 |
| MEF2C | 0.971458 | 1.07E-16 | 0.00E+00 | 1.55E-02 | 0.00E+00 | 2.77E-03 | | 0.01024008 |
| IL6 | 0.9596294 | 0.00E+00 | 0.00E+00 | 0.00E+00 | 4.56E-03 | 1.86E-04 | | 0.03562119 |
| CR1 | 0.9475295 | 7.81E-11 | 0.00E+00 | 1.33E-02 | 0.00E+00 | 1.83E-03 | | 0.0373621 |
| IFNB1 | 0.945531 | 0.00E+00 | 3.67E-11 | 9.39E-12 | 0.00E+00 | 3.55E-03 | | 0.05091466 |
| C8A | 0.9435178 | 2.37E-10 | 0.00E+00 | 0.00E+00 | 8.79E-03 | 7.00E-03 | | 0.04068781 |
| CXCL2 | 0.9376295 | 0.00E+00 | 0.00E+00 | 3.38E-02 | 3.98E-05 | 1.34E-03 | | 0.02722871 |
| CD34 | 0.9348198 | 7.76E-11 | 3.52E-02 | 8.99E-03 | 0.00E+00 | 3.02E-04 | | 0.02066681 |
| RRAD | 0.9326662 | 1.21E-02 | 2.79E-09 | 0.00E+00 | 0.00E+00 | 6.31E-03 | | 0.04894386 |

**Supplementary Table S5.** Top 10 most variable genes with respect to adverse effects.

|  | **logFC** | **AveExpr** | **t** | **P.Value** | **adj.P.Val** |
| --- | --- | --- | --- | --- | --- |
| **CXCL12** | -4143.341 | 5509.739 | -6.311505 | 0.000165 | 0.130034 |
| **A2M** | -2517.734 | 3237.298 | -5.013097 | 0.000819 | 0.19332 |
| **CD14** | -342.4033 | 308.8094 | -4.912786 | 0.000936 | 0.19332 |
| **TLR4** | -239.2156 | 269.6631 | -4.877139 | 0.000981 | 0.19332 |
| **LRP1** | -1066.312 | 1850.565 | -4.630522 | 0.001373 | 0.199439 |
| **CR1** | -259.861 | 112.4969 | -4.557533 | 0.001519 | 0.199439 |
| **C1QA** | -707.8877 | 593.4181 | -4.439016 | 0.001792 | 0.201757 |
| **BST1** | -79.80126 | 113.9475 | -4.258134 | 0.002317 | 0.222899 |
| **COLEC12** | -84.07576 | 131.8988 | -4.14973 | 0.002709 | 0.222899 |
| **MME** | -174.0912 | 154.0819 | -4.118996 | 0.002832 | 0.222899 |
| **LGMN** | -1269.282 | 2238.027 | -3.995628 | 0.003392 | 0.222899 |
| **TAB1** | -140.9525 | 541.2175 | -3.950148 | 0.003627 | 0.222899 |
| **ATF2** | -205.4777 | 763.4444 | -3.940918 | 0.003677 | 0.222899 |
| **PECAM1** | -1259.607 | 1769.643 | -3.879919 | 0.004025 | 0.224897 |
| **GADPH** | -8776.724 | 25846.99 | -3.82799 | 0.004349 | 0.224897 |
| **SERPING1** | -2366.512 | 3047.169 | -3.79542 | 0.004566 | 0.224897 |
| **S100B** | -159.8288 | 244.9244 | -3.733228 | 0.005014 | 0.230228 |
| **TNFSF12** | -403.9983 | 585.515 | -3.701571 | 0.005259 | 0.230228 |
| **POLR1B** | 219.8264 | 538.6344 | 3.496689 | 0.007186 | 0.257131 |
| **TNFSF13** | -94.83472 | 154.6013 | -3.490854 | 0.007251 | 0.257131 |
| **ICAM2** | -123.6485 | 180.9381 | -3.479105 | 0.007383 | 0.257131 |
| **SIGLEC1** | -371.386 | 434.64 | -3.467048 | 0.007522 | 0.257131 |
| **CD163** | -859.0631 | 661.4075 | -3.434963 | 0.007903 | 0.257131 |
| **NRP1** | -1085.829 | 1933.844 | -3.378513 | 0.008625 | 0.257131 |
| **VEGFC** | -133.0984 | 205.3094 | -3.370432 | 0.008734 | 0.257131 |
| **IL1RL2** | 50.78798 | 107.5713 | 3.316516 | 0.009497 | 0.257131 |
| **LRRN3** | -35.56273 | 46.83375 | -3.293136 | 0.00985 | 0.257131 |
| **FEZ1** | -181.0245 | 331.8688 | -3.281844 | 0.010025 | 0.257131 |
| **ENG** | -380.3616 | 785.1731 | -3.269133 | 0.010226 | 0.257131 |
| **CFI** | -75.36566 | 118.8781 | -3.266396 | 0.01027 | 0.257131 |
| **FCGR2B** | -349.0758 | 381.1425 | -3.26461 | 0.010299 | 0.257131 |
| **C7** | -262.3657 | 219.9044 | -3.217069 | 0.011095 | 0.257131 |
| **C4B** | -224.4663 | 213.6031 | -3.200462 | 0.011388 | 0.257131 |
| **MCAM** | -271.5146 | 498.9006 | -3.17385 | 0.011874 | 0.257131 |
| **C1QB** | -1122.225 | 1149.824 | -3.148862 | 0.012351 | 0.257131 |
| **GUSB** | -125.5982 | 389.0488 | -3.143152 | 0.012462 | 0.257131 |
| **NCF4** | -120.5044 | 200.5725 | -3.121313 | 0.012899 | 0.257131 |
| **C3AR1** | -115.7447 | 131.9994 | -3.085481 | 0.01365 | 0.257131 |
| **ITGB1** | -978.5757 | 3881.564 | -3.076292 | 0.01385 | 0.257131 |
| **C1R** | -3591.962 | 6686.856 | -3.071149 | 0.013963 | 0.257131 |
| **PRKCE** | -43.25887 | 99.11875 | -3.068436 | 0.014023 | 0.257131 |
| **SH2B2** | -63.74535 | 88.81875 | -3.066434 | 0.014068 | 0.257131 |
| **CD58** | 182.5471 | 839.5625 | 3.066312 | 0.01407 | 0.257131 |
| **MFGE8** | -1093.057 | 2115.004 | -3.053547 | 0.014358 | 0.257131 |
| **PDGFC** | -190.4903 | 298.3569 | -3.011285 | 0.015353 | 0.268257 |
| **CMKLR1** | -180.394 | 341.7319 | -2.996042 | 0.015729 | 0.268257 |
| **MEF2C** | -1043.06 | 569.0381 | -2.971583 | 0.016353 | 0.268257 |
| **CTSL** | -390.4037 | 618.9813 | -2.967791 | 0.016452 | 0.268257 |
| **ATG16L1** | 118.2871 | 450.6044 | 2.9591 | 0.016681 | 0.268257 |
| **F13A1** | -1849.986 | 2581.796 | -2.93447 | 0.017348 | 0.269062 |
| **MRC1** | -528.3286 | 547.3481 | -2.932102 | 0.017414 | 0.269062 |
| **CD209** | -162.2945 | 160.1338 | -2.850316 | 0.019843 | 0.300703 |
| **MST1R** | 162.391 | 489.2625 | 2.820514 | 0.020813 | 0.3055 |
| **TOLLIP** | 486.1197 | 1718.842 | 2.816851 | 0.020935 | 0.3055 |
| **HDAC3** | 173.1805 | 893.2263 | 2.771003 | 0.022532 | 0.32282 |
| **NLRP3** | -113.4827 | 83.99313 | -2.750104 | 0.0233 | 0.322871 |
| **TGFB2** | -60.9245 | 145.3569 | -2.739022 | 0.023719 | 0.322871 |
| **CDH5** | -161.0322 | 283.3181 | -2.737817 | 0.023765 | 0.322871 |
| **IL11** | 22.56396 | 34.60938 | 2.692985 | 0.02554 | 0.340366 |
| **CCL23** | -39.83755 | 27.04813 | -2.680131 | 0.026073 | 0.340366 |
| **CD99** | -2445.421 | 6277.105 | -2.673615 | 0.026348 | 0.340366 |
| **JAK1** | -141.6889 | 1001.321 | -2.653766 | 0.027203 | 0.342276 |
| **CD34** | -245.5088 | 207.9275 | -2.640597 | 0.027786 | 0.342276 |
| **CSF3R** | -267.6725 | 186.4463 | -2.631696 | 0.028187 | 0.342276 |
| **CD81** | -2547.674 | 12613.23 | -2.624252 | 0.028527 | 0.342276 |
| **BCL6** | -318.7439 | 1257.156 | -2.602393 | 0.029549 | 0.342276 |
| **NCAM1** | -217.9518 | 106.4994 | -2.598602 | 0.029731 | 0.342276 |
| **CXCL2** | -436.0857 | 208.8425 | -2.594073 | 0.029948 | 0.342276 |
| **ITGA1** | -193.1388 | 419.7513 | -2.580337 | 0.030619 | 0.342276 |
| **CD68** | -331.2732 | 527.7281 | -2.5759 | 0.030838 | 0.342276 |
| **PILRA** | -99.23555 | 225.3625 | -2.575876 | 0.03084 | 0.342276 |
| **TNFRSF17** | -41.0719 | 41.45875 | -2.556857 | 0.0318 | 0.342388 |
| **CD63** | -3209.55 | 10486.7 | -2.549736 | 0.032167 | 0.342388 |
| **CXCL3** | -74.34938 | 54.40813 | -2.53076 | 0.033166 | 0.342388 |
| **RIPK2** | 105.0873 | 382.8275 | 2.523625 | 0.03355 | 0.342388 |
| **STAT5B** | -160.7192 | 620.8256 | -2.514524 | 0.034046 | 0.342388 |
| **C6** | -45.7758 | 37.5425 | -2.513466 | 0.034105 | 0.342388 |
| **CXCL13** | -349.3978 | 282.0894 | -2.505335 | 0.034555 | 0.342388 |
| **JAM3** | -222.5797 | 420.2981 | -2.503856 | 0.034637 | 0.342388 |
| **IL8** | -4344.498 | 4299.304 | -2.501661 | 0.03476 | 0.342388 |
| **IL19** | -465.0745 | 96.18188 | -2.48965 | 0.03544 | 0.34384 |
| **LY96** | -136.7163 | 259.2869 | -2.483731 | 0.03578 | 0.34384 |
| **ADA** | -72.94791 | 152.1306 | -2.438531 | 0.038487 | 0.364329 |
| **TAL1** | -52.64117 | 71.01625 | -2.432917 | 0.038837 | 0.364329 |
| **CYBB** | -429.4666 | 485.6794 | -2.401148 | 0.040879 | 0.365076 |
| **CD207** | 322.1084 | 570.4156 | 2.397973 | 0.041089 | 0.365076 |
| **F12** | 103.7737 | 187.0975 | 2.391851 | 0.041497 | 0.365076 |
| **PIN1** | -40.93788 | 441.7988 | -2.369978 | 0.042987 | 0.365076 |
| **C2** | -108.4325 | 259.0206 | -2.352932 | 0.044185 | 0.365076 |
| **CXCL6** | -79.33682 | 34.4775 | -2.346912 | 0.044616 | 0.365076 |
| **LBP** | -32.60108 | 23.38125 | -2.346626 | 0.044637 | 0.365076 |
| **IFITM1** | -1018.927 | 2923.579 | -2.342947 | 0.044902 | 0.365076 |
| **FPR2** | -98.23098 | 60.8125 | -2.341001 | 0.045043 | 0.365076 |
| **FCGR2A** | -432.4632 | 486.6425 | -2.340827 | 0.045056 | 0.365076 |
| **CSF1** | -96.51776 | 260.2569 | -2.338207 | 0.045247 | 0.365076 |
| **TXNIP** | -3060.032 | 8801.471 | -2.337762 | 0.045279 | 0.365076 |
| **SLC11A1** | -125.4981 | 99.18938 | -2.325112 | 0.046212 | 0.365076 |
| **IL12RB2** | 31.85156 | 55.96313 | 2.323406 | 0.046339 | 0.365076 |
| **CDKN1A** | 1335.793 | 2884.873 | 2.320812 | 0.046533 | 0.365076 |
| **IL6** | -79.35532 | 36.56438 | -2.317413 | 0.046789 | 0.365076 |
| **TNFRSF10C** | -60.53331 | 52.89813 | -2.317363 | 0.046793 | 0.365076 |
| **RORA** | 1772.168 | 3813.811 | 2.310889 | 0.047283 | 0.365288 |
| **CXCL5** | -25.27869 | 18.835 | -2.288328 | 0.049034 | 0.375132 |
| **IL6ST** | -938.9982 | 3212.957 | -2.280385 | 0.049665 | 0.376309 |

**Supplementary Table S6.** 104 genes were found to be significantly differentially expressed in patients without adverse effects (AEs) versus a baseline of patients with AEs. A negative log fold change indicates that the gene is more highly expressed in patients with AEs relative to patients without AEs.

|  | **Pathway** | **External ID** | **Source** | **P value** | **FDR** |
| --- | --- | --- | --- | --- | --- |
| 1 | classical complement pathway | classicpathway | BioCarta | 0.00562056 | 1 |
| 2 | Transfer of LPS from LBP carrier to CD14 | R-HSA-166020 | Reactome | 0.01727307 | 1 |
| 3 | Complement Activation | WP545 | Wikipathways | 0.01770274 | 1 |
| 4 | Complement and Coagulation Cascades | WP558 | Wikipathways | 0.0269512 | 1 |
| 5 | Dengue-2 Interactions with Complement and Coagulation Cascades | WP3896 | Wikipathways | 0.03047909 | 1 |
| 6 | IL-6 signaling | None | INOH | 0.03313055 | 1 |
| 7 | TRAF6-mediated induction of TAK1 complex within TLR4 complex | R-HSA-937072 | Reactome | 0.04467303 | 1 |

**Supplementary Table S7.** 7 genes were found to be significantly differentially expressed in patients who experienced AEs relative to those who did not experience AE

| **Number** | **Pathway** | **FDR** | **Corrected P-Value** |
| --- | --- | --- | --- |
| 1 | TSLP | 1.69E-21 | 7.74E-18 |
| 2 | Thymic Stromal LymphoPoietin (TSLP) Signaling Pathway | 7.66E-18 | 1.76E-14 |
| 3 | IL-4 Signaling Pathway | 2.97E-17 | 4.55E-14 |
| 4 | IL4 | 1.31E-16 | 1.51E-13 |
| 5 | Th17 cell differentiation | 1.93E-14 | 1.77E-11 |
| 6 | IL12-mediated signaling events | 3.02E-14 | 2.07E-11 |
| 7 | AGE-RAGE pathway | 3.43E-14 | 2.07E-11 |
| 8 | EGFR Inhibitor Pathway, Pharmacodynamics | 3.89E-14 | 2.07E-11 |
| 9 | Pathways in cancer | 4.05E-14 | 2.07E-11 |
| 10 | Prolactin Signaling Pathway | 1.12E-13 | 4.66E-11 |
| 11 | IL2 | 1.12E-13 | 4.66E-11 |
| 12 | Glucocorticoid receptor regulatory network | 1.71E-13 | 6.54E-11 |
| 13 | Human T-cell leukemia virus 1 infection | 2.40E-13 | 8.48E-11 |
| 14 | Hepatitis B | 2.97E-13 | 9.52E-11 |
| 15 | Cytokine Signaling in Immune system | 3.11E-13 | 9.52E-11 |
| 16 | RANKL-RANK (Receptor activator of NFKB (ligand)) Signaling Pathway | 1.22E-12 | 3.49E-10 |
| 17 | PDGFR-beta pathway | 2.70E-12 | 7.29E-10 |
| 18 | Pathways in cancer | 4.14E-12 | 1.06E-09 |
| 19 | Acute myeloid leukemia | 4.63E-12 | 1.12E-09 |
| 20 | Prolactin signaling pathway | 7.10E-12 | 1.55E-09 |
| 21 | Prolactin | 7.10E-12 | 1.55E-09 |
| 22 | Photodynamic therapy-induced NF-kB survival signaling | 9.17E-12 | 1.91E-09 |
| 23 | IL6 | 1.06E-11 | 2.12E-09 |
| 24 | Oncostatin_M | 1.56E-11 | 2.98E-09 |
| 25 | TNF related weak inducer of apoptosis (TWEAK) Signaling Pathway | 2.53E-11 | 4.64E-09 |
| 26 | Tacrolimus/Cyclosporine Pathway, Pharmacodynamics | 3.96E-11 | 6.98E-09 |
| 27 | Signaling by Interleukins | 5.04E-11 | 8.34E-09 |
| 28 | Th1 and Th2 cell differentiation | 5.09E-11 | 8.34E-09 |
| 29 | IL5 | 6.85E-11 | 1.08E-08 |
| 30 | AGE-RAGE signaling pathway in diabetic complications | 8.59E-11 | 1.31E-08 |
| 31 | C-type lectin receptor signaling pathway | 1.22E-10 | 1.81E-08 |
| 32 | JAK-STAT | 1.40E-10 | 2.00E-08 |
| 33 | tpo signaling pathway | 2.62E-10 | 3.64E-08 |
| 34 | Viral carcinogenesis | 3.06E-10 | 4.14E-08 |
| 35 | miRNAs involvement in the immune response in sepsis | 4.12E-10 | 5.41E-08 |
| 36 | Oncostatin M Signaling Pathway | 4.54E-10 | 5.63E-08 |
| 37 | IL4-mediated signaling events | 4.54E-10 | 5.63E-08 |
| 38 | Osteoclast differentiation | 5.03E-10 | 6.08E-08 |
| 39 | Non-genomic actions of 1,25 dihydroxyvitamin D3 | 6.00E-10 | 7.06E-08 |
| 40 | Adipogenesis | 6.26E-10 | 7.18E-08 |
| 41 | Downstream signal transduction | 6.99E-10 | 7.83E-08 |
| 42 | KitReceptor | 7.17E-10 | 7.84E-08 |
| 43 | Toll-like Receptor Signaling | 8.33E-10 | 8.89E-08 |
| 44 | TNFalpha | 1.03E-09 | 1.07E-07 |
| 45 | Leptin signaling pathway | 1.19E-09 | 1.21E-07 |
| 46 | Brain-Derived Neurotrophic Factor (BDNF) signaling pathway | 1.22E-09 | 1.22E-07 |
| 47 | il22 soluble receptor signaling pathway | 1.34E-09 | 1.31E-07 |
| 48 | IL9 | 2.00E-09 | 1.92E-07 |
| 49 | IL23-mediated signaling events | 2.12E-09 | 1.95E-07 |
| 50 | GMCSF-mediated signaling events | 2.12E-09 | 1.95E-07 |
| 51 | Signaling events mediated by TCPTP | 2.44E-09 | 2.20E-07 |
| 52 | IL-5 Signaling Pathway | 3.20E-09 | 2.82E-07 |
| 53 | IL-2 Signaling Pathway | 4.13E-09 | 3.57E-07 |
| 54 | Signaling by SCF-KIT | 4.66E-09 | 3.96E-07 |
| 55 | Transcriptional misregulation in cancer | 7.02E-09 | 5.86E-07 |
| 56 | IL6-mediated signaling events | 7.40E-09 | 6.07E-07 |
| 57 | EGFR1 | 8.72E-09 | 7.02E-07 |
| 58 | IL-9 Signaling Pathway | 9.59E-09 | 7.59E-07 |
| 59 | Angiopoietin receptor Tie2-mediated signaling | 1.02E-08 | 7.80E-07 |
| 60 | JAK STAT MolecularVariation 2 | 1.02E-08 | 7.80E-07 |
| 61 | TNF signaling pathway | 1.13E-08 | 8.50E-07 |
| 62 | Epstein-Barr virus infection | 1.21E-08 | 8.93E-07 |
| 63 | Signaling by cytosolic FGFR1 fusion mutants | 1.23E-08 | 8.97E-07 |
| 64 | Signaling by PDGF | 1.52E-08 | 1.07E-06 |
| 65 | IL2-mediated signaling events | 1.52E-08 | 1.07E-06 |
| 66 | Growth hormone receptor signaling | 1.56E-08 | 1.08E-06 |
| 67 | FGF signaling pathway | 1.83E-08 | 1.25E-06 |
| 68 | Immune System | 1.90E-08 | 1.28E-06 |
| 69 | inhibition of cellular proliferation by gleevec | 2.93E-08 | 1.95E-06 |
| 70 | Ebola Virus Pathway on Host | 3.09E-08 | 2.02E-06 |
| 71 | Measles | 3.23E-08 | 2.09E-06 |
| 72 | RANKL | 3.55E-08 | 2.26E-06 |
| 73 | VEGFA-VEGFR2 Signaling Pathway | 3.78E-08 | 2.38E-06 |
| 74 | RAC1-PAK1-p38-MMP2 Pathway | 4.93E-08 | 3.06E-06 |
| 75 | IL-7 Signaling Pathway | 5.06E-08 | 3.09E-06 |
| 76 | EPO Receptor Signaling | 5.97E-08 | 3.61E-06 |
| 77 | Alternative NF-kappaB pathway | 6.22E-08 | 3.71E-06 |
| 78 | IL-7 | 7.00E-08 | 4.12E-06 |
| 79 | Pertussis | 8.67E-08 | 5.04E-06 |
| 80 | Interleukin-1 processing | 1.09E-07 | 6.24E-06 |
| 81 | IL17 signaling pathway | 1.25E-07 | 7.00E-06 |
| 82 | FGFR1 mutant receptor activation | 1.25E-07 | 7.00E-06 |
| 83 | White fat cell differentiation | 1.43E-07 | 7.80E-06 |
| 84 | Osteopontin-mediated events | 1.43E-07 | 7.80E-06 |
| 85 | EPO signaling pathway | 1.62E-07 | 8.66E-06 |
| 86 | bioactive peptide induced signaling pathway | 1.62E-07 | 8.66E-06 |
| 87 | CXCR4-mediated signaling events | 1.72E-07 | 9.05E-06 |
| 88 | ApoE and miR-146 in inflammation and atherosclerosis | 1.74E-07 | 9.06E-06 |
| 89 | CD40/CD40L signaling | 1.84E-07 | 9.48E-06 |
| 90 | IL-17 signaling pathway | 2.40E-07 | 1.22E-05 |
| 91 | Kaposi sarcoma-associated herpesvirus infection | 2.60E-07 | 1.30E-05 |
| 92 | Type II interferon signaling (IFNG) | 2.61E-07 | 1.30E-05 |
| 93 | Signaling by FGFR1 in disease | 2.91E-07 | 1.44E-05 |
| 94 | B Cell Receptor Signaling Pathway | 3.12E-07 | 1.51E-05 |
| 95 | JAK STAT MolecularVariation 1 | 3.12E-07 | 1.51E-05 |
| 96 | CLEC7A (Dectin-1) signaling | 3.60E-07 | 1.72E-05 |
| 97 | Toll-like Receptor Signaling Pathway | 3.81E-07 | 1.80E-05 |
| 98 | Gastrin | 3.98E-07 | 1.87E-05 |
| 99 | JAK-STAT-Core | 4.20E-07 | 1.95E-05 |
| 100 | IL-5 signaling | 5.11E-07 | 2.32E-05 |
| 101 | Signaling by Leptin | 5.11E-07 | 2.32E-05 |
| 102 | RAGE | 5.84E-07 | 2.63E-05 |
| 103 | Signaling by FGFR in disease | 6.98E-07 | 3.11E-05 |
| 104 | IL-3 Signaling Pathway | 8.27E-07 | 3.65E-05 |
| 105 | Osteopontin Signaling | 8.83E-07 | 3.79E-05 |
| 106 | Development of pulmonary dendritic cells and macrophage subsets | 8.83E-07 | 3.79E-05 |
| 107 | DEx/H-box helicases activate type I IFN and inflammatory cytokines production | 8.83E-07 | 3.79E-05 |
| 108 | Cytosolic sensors of pathogen-associated DNA | 9.74E-07 | 4.14E-05 |
| 109 | mechanism of gene regulation by peroxisome proliferators via ppara | 1.05E-06 | 4.44E-05 |
| 110 | Atypical NF-kappaB pathway | 1.12E-06 | 4.67E-05 |
| 111 | PDGFR-beta signaling pathway | 1.14E-06 | 4.67E-05 |
| 112 | keratinocyte differentiation | 1.14E-06 | 4.67E-05 |
| 113 | Interferon type I signaling pathways | 1.23E-06 | 4.95E-05 |
| 114 | IL1 | 1.23E-06 | 4.95E-05 |
| 115 | IL-1 signaling pathway | 1.32E-06 | 5.24E-05 |
| 116 | TGF-beta Receptor Signaling | 1.32E-06 | 5.24E-05 |
| 117 | Interleukin-4 and 13 signaling | 1.40E-06 | 5.50E-05 |
| 118 | Kit receptor signaling pathway | 1.76E-06 | 6.84E-05 |
| 119 | Regulation of toll-like receptor signaling pathway | 2.04E-06 | 7.88E-05 |
| 120 | LPA receptor mediated events | 2.29E-06 | 8.77E-05 |
| 121 | Regulation of retinoblastoma protein | 2.44E-06 | 9.27E-05 |
| 122 | ncRNAs involved in STAT3 signaling in hepatocellular carcinoma | 2.51E-06 | 9.36E-05 |
| 123 | Activation of NF-kappaB in B cells | 2.51E-06 | 9.36E-05 |
| 124 | CD209 (DC-SIGN) signaling | 2.97E-06 | 0.000110108 |
| 125 | BCR signaling pathway | 3.12E-06 | 0.000114563 |
| 126 | JAK-STAT signaling pathway | 3.77E-06 | 0.000135312 |
| 127 | Necroptosis | 3.77E-06 | 0.000135312 |
| 128 | EGF-EGFR Signaling Pathway | 3.77E-06 | 0.000135312 |
| 129 | Chemokine signaling pathway | 4.13E-06 | 0.0001465 |
| 130 | Leishmaniasis | 4.15E-06 | 0.0001465 |
| 131 | Pancreatic cancer | 4.62E-06 | 0.000162028 |
| 132 | Chronic myeloid leukemia | 4.88E-06 | 0.000168291 |
| 133 | Viral Acute Myocarditis | 4.88E-06 | 0.000168291 |
| 134 | EBV LMP1 signaling | 5.42E-06 | 0.000182764 |
| 135 | RIP-mediated NFkB activation via ZBP1 | 5.42E-06 | 0.000182764 |
| 136 | IL11 | 5.42E-06 | 0.000182764 |
| 137 | C-type lectin receptors (CLRs) | 6.94E-06 | 0.000232396 |
| 138 | C-MYB transcription factor network | 7.63E-06 | 0.000253708 |
| 139 | Cellular Senescence | 7.81E-06 | 0.000257975 |
| 140 | TRAF6 mediated NF-kB activation | 7.93E-06 | 0.00025804 |
| 141 | IL27-mediated signaling events | 7.93E-06 | 0.00025804 |
| 142 | Chemokine signaling pathway | 8.02E-06 | 0.000259136 |
| 143 | Apoptosis | 8.37E-06 | 0.000263105 |
| 144 | MyD88 cascade initiated on plasma membrane | 8.37E-06 | 0.000263105 |
| 145 | Toll Like Receptor 10 (TLR10) Cascade | 8.37E-06 | 0.000263105 |
| 146 | Toll Like Receptor 5 (TLR5) Cascade | 8.37E-06 | 0.000263105 |
| 147 | TRAF6 mediated induction of NFkB and MAP kinases upon TLR7/8 or 9 activation | 8.76E-06 | 0.000273488 |
| 148 | pdgf signaling pathway | 8.91E-06 | 0.000274439 |
| 149 | ZBP1(DAI) mediated induction of type I IFNs | 8.91E-06 | 0.000274439 |
| 150 | Pancreatic adenocarcinoma pathway | 9.16E-06 | 0.000280354 |
| 151 | Toll Like Receptor 7/8 (TLR7/8) Cascade | 9.58E-06 | 0.000289248 |
| 152 | MyD88 dependent cascade initiated on endosome | 9.58E-06 | 0.000289248 |
| 153 | TWEAK | 9.97E-06 | 0.0002971 |
| 154 | IL3-mediated signaling events | 9.97E-06 | 0.0002971 |
| 155 | T-Cell antigen Receptor (TCR) Signaling Pathway | 1.05E-05 | 0.000309564 |
| 156 | TNF alpha Signaling Pathway | 1.09E-05 | 0.000321086 |
| 157 | the information processing pathway at the ifn beta enhancer | 1.11E-05 | 0.00032473 |
| 158 | NF-kappa B signaling pathway | 1.14E-05 | 0.000328705 |
| 159 | Toll Like Receptor 9 (TLR9) Cascade | 1.14E-05 | 0.000328705 |
| 160 | Downstream signaling events of B Cell Receptor (BCR) | 1.23E-05 | 0.000351502 |
| 161 | IL2 signaling events mediated by STAT5 | 1.23E-05 | 0.000351502 |
| 162 | Toll Like Receptor 3 (TLR3) Cascade | 1.29E-05 | 0.000356666 |
| 163 | MyD88:Mal cascade initiated on plasma membrane | 1.29E-05 | 0.000356666 |
| 164 | Toll Like Receptor TLR1:TLR2 Cascade | 1.29E-05 | 0.000356666 |
| 165 | Toll Like Receptor TLR6:TLR2 Cascade | 1.29E-05 | 0.000356666 |
| 166 | Toll Like Receptor 2 (TLR2) Cascade | 1.29E-05 | 0.000356666 |
| 167 | IL12 signaling mediated by STAT4 | 1.50E-05 | 0.000408784 |
| 168 | TAK1 activates NFkB by phosphorylation and activation of IKKs complex | 1.50E-05 | 0.000408784 |
| 169 | TRIF(TICAM1)-mediated TLR4 signaling | 1.51E-05 | 0.000408784 |
| 170 | MyD88-independent TLR4 cascade | 1.51E-05 | 0.000408784 |
| 171 | Prion disease pathway | 1.65E-05 | 0.000443431 |
| 172 | Toll-like receptor signaling pathway | 1.70E-05 | 0.000453666 |
| 173 | ErbB1 downstream signaling | 1.83E-05 | 0.000486333 |
| 174 | IL1-mediated signaling events | 2.16E-05 | 0.000568614 |
| 175 | Senescence-Associated Secretory Phenotype (SASP) | 2.20E-05 | 0.000576761 |
| 176 | Toxoplasmosis | 2.36E-05 | 0.000615332 |
| 177 | Intracellular Signalling Through Adenosine Receptor A2b and Adenosine | 2.54E-05 | 0.000651778 |
| 178 | Intracellular Signalling Through Adenosine Receptor A2a and Adenosine | 2.54E-05 | 0.000651778 |
| 179 | Selenium Metabolism and Selenoproteins | 2.54E-05 | 0.000651778 |
| 180 | MAPK Signaling Pathway | 2.87E-05 | 0.000731618 |
| 181 | Signaling by Receptor Tyrosine Kinases | 3.02E-05 | 0.000765713 |
| 182 | IFN-gamma pathway | 3.45E-05 | 0.000868922 |
| 183 | IL-6 signaling pathway | 3.70E-05 | 0.000928075 |
| 184 | Toll Like Receptor 4 (TLR4) Cascade | 3.74E-05 | 0.000931727 |
| 185 | Interleukin-11 Signaling Pathway | 3.97E-05 | 0.000979948 |
| 186 | BCR | 3.97E-05 | 0.000979948 |
| 187 | Relaxin signaling pathway | 4.09E-05 | 0.001004657 |
| 188 | PDGF | 4.25E-05 | 0.001036706 |
| 189 | TNF receptor signaling pathway | 4.54E-05 | 0.001102084 |
| 190 | IL-2 signaling | 4.70E-05 | 0.001130638 |
| 191 | IL-3 signaling | 4.70E-05 | 0.001130638 |
| 192 | Fluid shear stress and atherosclerosis | 5.32E-05 | 0.001271453 |
| 193 | Cocaine addiction | 5.49E-05 | 0.001306028 |
| 194 | Protein alkylation leading to liver fibrosis | 5.84E-05 | 0.001380873 |
| 195 | Vitamin B12 Metabolism | 6.20E-05 | 0.001443417 |
| 196 | Regulation of Androgen receptor activity | 6.20E-05 | 0.001443417 |
| 197 | IL3 | 6.20E-05 | 0.001443417 |
| 198 | NIK-->noncanonical NF-kB signaling | 6.27E-05 | 0.001445577 |
| 199 | Dectin-1 mediated noncanonical NF-kB signaling | 6.27E-05 | 0.001445577 |
| 200 | Signaling events mediated by Stem cell factor receptor (c-Kit) | 6.57E-05 | 0.001507332 |
| 201 | MAPK signaling pathway | 6.83E-05 | 0.001559794 |
| 202 | Signaling events mediated by PTP1B | 6.96E-05 | 0.001576796 |
| 203 | Non-alcoholic fatty liver disease (NAFLD) | 6.97E-05 | 0.001576796 |
| 204 | RAC1 signaling pathway | 7.36E-05 | 0.001655334 |
| 205 | Legionellosis | 7.77E-05 | 0.001740584 |
| 206 | Toll-Like Receptors Cascades | 7.93E-05 | 0.001766734 |
| 207 | Supression of HMGB1 mediated inflammation by THBD | 8.05E-05 | 0.001768296 |
| 208 | Role Altered Glycolysation of MUC1 in Tumour Microenvironment | 8.05E-05 | 0.001768296 |
| 209 | Hepatitis C | 8.13E-05 | 0.001768296 |
| 210 | Nonalcoholic fatty liver disease | 8.13E-05 | 0.001768296 |
| 211 | Hepatitis C and Hepatocellular Carcinoma | 8.21E-05 | 0.001768296 |
| 212 | DDX58/IFIH1-mediated induction of interferon-alpha/beta | 8.21E-05 | 0.001768296 |
| 213 | Signaling events mediated by HDAC Class I | 8.21E-05 | 0.001768296 |
| 214 | JAK STAT pathway and regulation | 8.51E-05 | 0.001825099 |
| 215 | mapkinase signaling pathway | 8.65E-05 | 0.001847357 |
| 216 | Sudden Infant Death Syndrome (SIDS) Susceptibility Pathways | 8.98E-05 | 0.001907578 |
| 217 | Notch | 9.12E-05 | 0.001928273 |
| 218 | MET in type 1 papillary renal cell carcinoma | 9.60E-05 | 0.00202027 |
| 219 | Mammary gland development pathway - Involution (Stage 4 of 4) | 0.00010054 | 0.002086234 |
| 220 | Growth hormone signaling | 0.00010054 | 0.002086234 |
| 221 | Interleukin-27 signaling | 0.00010054 | 0.002086234 |
| 222 | Interleukin-1 signaling | 0.0001009 | 0.002086234 |
| 223 | NOD-like receptor signaling pathway | 0.00011112 | 0.002280641 |
| 224 | Fc-epsilon receptor I signaling in mast cells | 0.0001113 | 0.002280641 |
| 225 | Endochondral Ossification | 0.00011675 | 0.00238169 |
| 226 | Influenza A | 0.000119 | 0.002416765 |
| 227 | Validated nuclear estrogen receptor alpha network | 0.00012237 | 0.002439404 |
| 228 | IL-1 NFkB | 0.00012237 | 0.002439404 |
| 229 | epo signaling pathway | 0.00012277 | 0.002439404 |
| 230 | Regulated proteolysis of p75NTR | 0.00012277 | 0.002439404 |
| 231 | Interleukin-6 signaling | 0.00012277 | 0.002439404 |
| 232 | Non-small cell lung cancer | 0.00013414 | 0.002619969 |
| 233 | Folate Metabolism | 0.00013414 | 0.002619969 |
| 234 | Human Thyroid Stimulating Hormone (TSH) signaling pathway | 0.00013414 | 0.002619969 |
| 235 | Non-small cell lung cancer | 0.00013414 | 0.002619969 |
| 236 | Tuberculosis | 0.00013894 | 0.00270225 |
| 237 | TNF | 0.00014029 | 0.002716966 |
| 238 | Cellular responses to stress | 0.0001411 | 0.002721169 |
| 239 | Epithelial cell signaling in Helicobacter pylori infection | 0.00014662 | 0.002791646 |
| 240 | Regulation of Telomerase | 0.00014662 | 0.002791646 |
| 241 | il 3 signaling pathway | 0.00014718 | 0.002791646 |
| 242 | Interleukin-2 signaling | 0.00014718 | 0.002791646 |
| 243 | Adipocytokine signaling pathway | 0.00015313 | 0.002880652 |
| 244 | TLR NFkB | 0.00015313 | 0.002880652 |
| 245 | Ras Signaling | 0.00015788 | 0.002957792 |
| 246 | B cell receptor signaling pathway | 0.00015983 | 0.002958189 |
| 247 | AP-1 transcription factor network | 0.00015983 | 0.002958189 |
| 248 | Interferon alpha/beta signaling | 0.00015983 | 0.002958189 |
| 249 | Herpes simplex infection | 0.00016121 | 0.002959763 |
| 250 | EPO signaling | 0.00016121 | 0.002959763 |
| 251 | TFs Regulate miRNAs related to cardiac hypertrophy | 0.00017379 | 0.003115913 |
| 252 | Estrogen Receptor Pathway | 0.00017379 | 0.003115913 |
| 253 | Transcriptional cascade regulating adipogenesis | 0.00017379 | 0.003115913 |
| 254 | the 41bb-dependent immune response | 0.00017379 | 0.003115913 |
| 255 | il 6 signaling pathway | 0.00017379 | 0.003115913 |
| 256 | NF-kB is activated and signals survival | 0.00017379 | 0.003115913 |
| 257 | PKMTs methylate histone lysines | 0.00018107 | 0.003233875 |
| 258 | Validated targets of C-MYC transcriptional repression | 0.00018853 | 0.003354159 |
| 259 | Prolactin receptor signaling | 0.00020256 | 0.003548704 |
| 260 | IL5-mediated signaling events | 0.00020256 | 0.003548704 |
| 261 | erythropoietin mediated neuroprotection through nf-kb | 0.00020256 | 0.003548704 |
| 262 | il 2 signaling pathway | 0.00020256 | 0.003548704 |
| 263 | Interleukin-1 family signaling | 0.00021212 | 0.003702092 |
| 264 | Transcriptional regulation of white adipocyte differentiation | 0.00023351 | 0.004029362 |
| 265 | Quercetin and Nf-kB- AP-1 Induced Cell Apoptosis | 0.00023351 | 0.004029362 |
| 266 | il-7 signal transduction | 0.00023351 | 0.004029362 |
| 267 | Selenium Micronutrient Network | 0.00026488 | 0.004549426 |
| 268 | p75NTR signals via NF-kB | 0.00026662 | 0.004549426 |
| 269 | cadmium induces dna synthesis and proliferation in macrophages | 0.00026662 | 0.004549426 |
| 270 | ErbB signaling pathway | 0.00028418 | 0.004831029 |
| 271 | Salmonella infection | 0.00029416 | 0.004982281 |
| 272 | Androgen receptor signaling pathway | 0.00032546 | 0.005492184 |
| 273 | Cellular responses to external stimuli | 0.00033064 | 0.005559061 |
| 274 | atm signaling pathway | 0.00033932 | 0.005663493 |
| 275 | mets affect on macrophage differentiation | 0.00033932 | 0.005663493 |
| 276 | ErbB Signaling Pathway | 0.00035884 | 0.005967686 |
| 277 | Interleukin-12 family signaling | 0.00037889 | 0.006278286 |
| 278 | Interferon gamma signaling | 0.00038228 | 0.006302781 |
| 279 | Ras signaling pathway | 0.00038311 | 0.006302781 |
| 280 | Prostate cancer | 0.00041924 | 0.006870227 |
| 281 | egf signaling pathway | 0.0004206 | 0.006870227 |
| 282 | HIF-1 signaling pathway | 0.00045844 | 0.007461833 |
| 283 | TCR | 0.00046379 | 0.007479927 |
| 284 | nf-kb signaling pathway | 0.00046444 | 0.007479927 |
| 285 | IFN alpha signaling | 0.00046444 | 0.007479927 |
| 286 | T cell receptor signaling pathway | 0.00047201 | 0.007548867 |
| 287 | Neural Crest Differentiation | 0.00047201 | 0.007548867 |
| 288 | Chagas disease (American trypanosomiasis) | 0.00048584 | 0.007743012 |
| 289 | Diseases of signal transduction | 0.0004932 | 0.007833218 |
| 290 | Transcription factor regulation in adipogenesis | 0.00051041 | 0.007968685 |
| 291 | The human immune response to tuberculosis | 0.00051041 | 0.007968685 |
| 292 | Extracellular vesicles in the crosstalk of cardiac cells | 0.00051041 | 0.007968685 |
| 293 | Vitamin D in inflammatory diseases | 0.00051041 | 0.007968685 |
| 294 | Interleukin-6 family signaling | 0.00051041 | 0.007968685 |
| 295 | Senescence and Autophagy in Cancer | 0.00054374 | 0.008460236 |
| 296 | Estrogen signaling pathway | 0.00055851 | 0.008579413 |
| 297 | Canonical NF-kappaB pathway | 0.00055851 | 0.008579413 |
| 298 | growth hormone signaling pathway | 0.00055851 | 0.008579413 |
| 299 | Insulin resistance | 0.00055888 | 0.008579413 |
| 300 | DNA Damage Response (only ATM dependent) | 0.0006059 | 0.00925172 |
| 301 | Physiological and Pathological Hypertrophy of the Heart | 0.00060872 | 0.00925172 |
| 302 | IL1 and megakaryocytes in obesity | 0.00060872 | 0.00925172 |
| 303 | Vemurafenib Pathway, Pharmacodynamics | 0.00066104 | 0.009948119 |
| 304 | update your name in edit mode | 0.00066104 | 0.009948119 |
| 305 | Differentiation of white and brown adipocyte | 0.00066104 | 0.009948119 |
| 306 | Interleukin-7 signaling | 0.00071547 | 0.010732001 |
| 307 | Innate Immune System | 0.00072848 | 0.010891616 |
| 308 | Neurotrophin signaling pathway | 0.00076197 | 0.011355325 |
| 309 | Disease | 0.00085213 | 0.012657921 |
| 310 | Signaling by the B Cell Receptor (BCR) | 0.00092044 | 0.013628521 |
| 311 | CD4 T cell receptor signaling | 0.00098487 | 0.014535512 |
| 312 | ErbB4 signaling events | 0.00101896 | 0.01494252 |
| 313 | amb2 Integrin signaling | 0.00101896 | 0.01494252 |
| 314 | TGF-beta Signaling Pathway | 0.00102937 | 0.015047153 |
| 315 | Initiation of transcription and translation elongation at the HIV-1 LTR | 0.00108588 | 0.015822886 |
| 316 | Apoptosis | 0.00112216 | 0.016299763 |
| 317 | Mammary gland development pathway - Pregnancy and lactation (Stage 3 of 4) | 0.00115488 | 0.016617175 |
| 318 | Resistin as a regulator of inflammation | 0.00115488 | 0.016617175 |
| 319 | role of egf receptor transactivation by gpcrs in cardiac hypertrophy | 0.00115488 | 0.016617175 |
| 320 | Signaling of Hepatocyte Growth Factor Receptor | 0.00122592 | 0.017584347 |
| 321 | AndrogenReceptor | 0.001297 | 0.018402836 |
| 322 | HIV-1 Nef: Negative effector of Fas and TNF-alpha | 0.00129902 | 0.018402836 |
| 323 | toll-like receptor pathway | 0.00129902 | 0.018402836 |
| 324 | Signaling mediated by p38-alpha and p38-beta | 0.00129902 | 0.018402836 |
| 325 | CRH | 0.00137417 | 0.019407494 |
| 326 | Validated transcriptional targets of AP1 family members Fra1 and Fra2 | 0.00145135 | 0.020310103 |
| 327 | signal transduction through il1r | 0.00145135 | 0.020310103 |
| 328 | IL2 signaling events mediated by PI3K | 0.00145135 | 0.020310103 |
| 329 | Fc epsilon receptor (FCERI) signaling | 0.00148817 | 0.020761982 |
| 330 | Interleukin-2 family signaling | 0.00153057 | 0.021288871 |
| 331 | Human papillomavirus infection | 0.00158367 | 0.021960807 |
| 332 | ErbB2/ErbB3 signaling events | 0.00169509 | 0.023435102 |
| 333 | Interferon Signaling | 0.00172737 | 0.023809708 |
| 334 | Cellular senescence | 0.00179071 | 0.024608922 |
| 335 | FOXA1 transcription factor network | 0.00204827 | 0.027980833 |
| 336 | Interleukin-3, 5 and GM-CSF signaling | 0.00204827 | 0.027980833 |
| 337 | FOXA2 and FOXA3 transcription factor networks | 0.00214156 | 0.029168476 |
| 338 | Aryl Hydrocarbon Receptor | 0.00223684 | 0.03028648 |
| 339 | BDNF | 0.00223684 | 0.03028648 |
| 340 | NO-cGMP-PKG mediated Neuroprotection | 0.00233411 | 0.031510453 |
| 341 | Ceramide signaling pathway | 0.00243335 | 0.032658077 |
| 342 | il-2 receptor beta chain in t cell activation | 0.00243335 | 0.032658077 |
| 343 | Hematopoietic Stem Cell Differentiation | 0.00253456 | 0.033818649 |
| 344 | Calcineurin-regulated NFAT-dependent transcription in lymphocytes | 0.00253456 | 0.033818649 |
| 345 | TNFR2 non-canonical NF-kB pathway | 0.00263773 | 0.035093297 |
| 346 | Downstream TCR signaling | 0.00274287 | 0.036386566 |
| 347 | Apoptosis-related network due to altered Notch3 in ovarian cancer | 0.00295899 | 0.039140506 |
| 348 | t cell receptor signaling pathway | 0.00318288 | 0.04198109 |
| 349 | cAMP signaling pathway | 0.00332831 | 0.043773474 |
| 350 | RIG-I-like Receptor Signaling | 0.0037763 | 0.049523536 |

**Supplementary Table S8.** 350 pathways were found to be significant in the network built examining genes one step upstream of the differentially expressed genes in CR relative to IR.

| **Number** | **Pathway** | **FDR** | **Corrected P-Value** |
| --- | --- | --- | --- |
| 1 | Type II interferon signaling (IFNG) | 7.89E-06 | 0.0353974 |
| 2 | RANKL-RANK (Receptor activator of NFKB (ligand)) Signaling Pathway | 1.76E-05 | 0.0353974 |
| 3 | IL4 | 2.31E-05 | 0.0353974 |
| 4 | Glucocorticoid receptor regulatory network | 3.74E-05 | 0.042955371 |

**Supplementary Table S9.** 4 pathways were found to be significant in the network built examining genes one step upstream of the differentially expressed genes in patients with AEs relative to those that did not experience AEs. Three of these pathways overlapped with those identified in CR relative to IR.

| ABCF1 |
| --- |
| AGK |
| ALAS1 |
| AMMECR1L |
| CC2D1B |
| CNOT10 |
| CNOT4 |
| COG7 |
| DDX50 |
| DHX16 |
| DNAJC14 |
| EDC3 |
| EIF2B4 |
| ERCC3 |
| FCF1 |
| G6PD |
| GPATCH3 |
| GUSB |
| HDAC3 |
| HPRT1 |
| MRPS5 |
| MTMR14 |
| NOL7 |
| NUBP1 |
| POLR2A |
| PPIA |
| PRPF38A |
| SAP130 |
| SDHA |
| SF3A3 |
| TBP |
| TLK2 |
| TMUB2 |
| TRIM39 |
| TUBB |
| USP39 |
| ZC3H14 |
| ZKSCAN5 |
| ZNF143 |
| ZNF346 |

**Supplemental Table S10**. Nanostring Housekeeping Control Genes
