# Supplementary material for: Transcriptomic analysis identifies differences in gene expression in actinic keratoses after treatment with imiquimod and between responders and non responders
Source: Sci Rep. 2021 Apr 22;11:8775. doi: 10.1038/s41598-021-88424-z (PMC8062619; doi:10.1038/s41598-021-88424-z)
Supplement: Supplementary file 2 — Supplementary Information 2. [file 41598_2021_88424_MOESM2_ESM.docx]

**Supplementary Figure**

Transcriptomic analysis identifies differences in gene expression in actinic keratoses after treatment with imiquimod and between responders and non responders

Short Title: Transcriptomic analysis of actinic keratoses

Megan H. Trager BA*^1^, Emanuelle Rizk BA*^2^, Sharon Rose MD*^3^, Kuixi Zhu MSc*^4^, Branden Lau BS^4^, Ben Fullerton BA^2^, Jaya Pradhan MD^5^, Michael Moore BS^6^, Ayush C. Srivastava MD^2^, Giselle Singer BS^3^, Robyn Gartrell MD^7^, Rui Chang PhD*^4^, Larisa J. Geskin MD*^1^, Yvonne M. Saenger MD*^2^, Gary Goldenberg MD*^3^

**
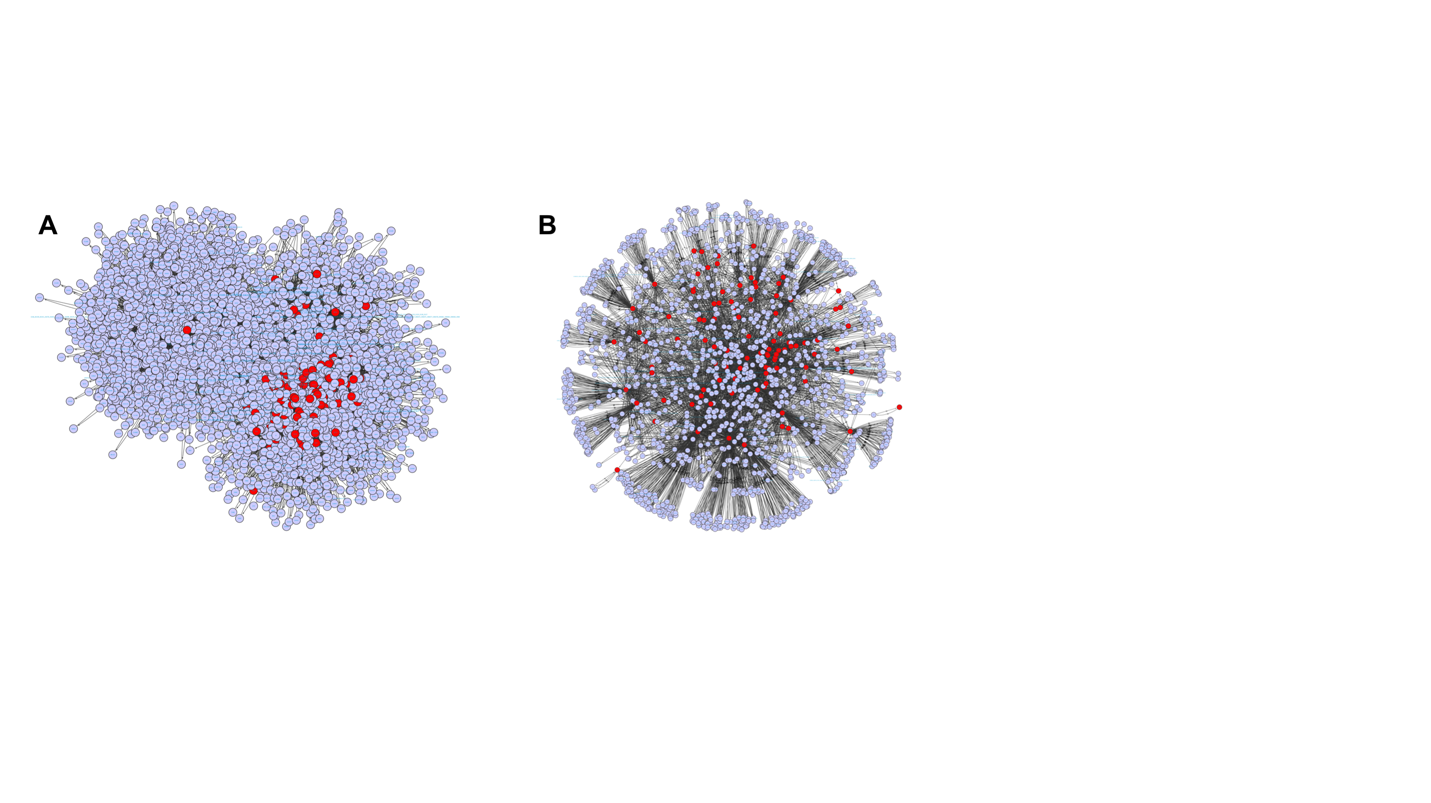
**

**Supplementary Figure S1.** Network analysis for comparison of (A) IR versus CR and (B) patients with AEs to patients without AEs. Red points are genes that are differentially expressed based on transcriptomic analyses. Surrounding points are neighboring pathways within two steps of red points. Figure was generated using Cytoscape 3.2.4 software: https://cytoscape.org
